# A diseasome cluster-based drug repurposing of soluble guanylate cyclase activators from smooth muscle relaxation to direct neuroprotection

**DOI:** 10.1038/s41540-017-0039-7

**Published:** 2018-02-05

**Authors:** Friederike Langhauser, Ana I. Casas, Vu-Thao-Vi Dao, Emre Guney, Jörg Menche, Eva Geuss, Pamela W. M. Kleikers, Manuela G. López, Albert-L. Barabási, Christoph Kleinschnitz, Harald H. H. W. Schmidt

**Affiliations:** 1 Department of Neurology, University Hospital Würzburg, Josef-Schneider-Straße 11, 97080 Würzburg, Germany; 2 Department of Neurology, University Hospital Essen, Hufelandstraße 55, D-45147 Essen, Germany; 3 Department of Pharmacology and Personalised Medicine, CARIM, FHML, Maastricht University, Universiteitssingel 50, 6229 ER Maastricht, The Netherlands; 4 Departamento de Farmacología, Facultad de Medicina, Universidad Autónoma de Madrid, Arzobispo Morcillo s/n, 28029 Madrid, Spain; 5 Center for Complex Network Research (CCNR) and Department of Physics, Northeastern University, Boston, MA 02115 USA; 6 Center for Cancer Systems Biology (CCSB) and Department of Cancer Biology, Dana-Farber Cancer Institute, Harvard Medical School, Boston, MA 02215 USA; 7 CeMM Research Center for Molecular Medicine of the Austrian Academy of Sciences, Lazarettgasse 14 AKH BT25.3, 1090 Vienna, Austria; 8 Department of Medicine, Brigham and Women’s Hospital, Harvard Medical School, Boston, MA 02115 USA; 9 Center for Network Science, Central European University, Nador u. 9, 1051 Budapest, Hungary

## Abstract

Network medicine utilizes common genetic origins, markers and co-morbidities to uncover mechanistic links between diseases. These links can be summarized in the diseasome, a comprehensive network of disease–disease relationships and clusters. The diseasome has been influential during the past decade, although most of its links are not followed up experimentally. Here, we investigate a high prevalence unmet medical need cluster of disease phenotypes linked to cyclic GMP. Hitherto, the central cGMP-forming enzyme, soluble guanylate cyclase (sGC), has been targeted pharmacologically exclusively for smooth muscle modulation in cardiology and pulmonology. Here, we examine the disease associations of sGC in a non-hypothesis based manner in order to identify possibly previously unrecognized clinical indications. Surprisingly, we find that sGC, is closest linked to neurological disorders, an application that has so far not been explored clinically. Indeed, when investigating the neurological indication of this cluster with the highest unmet medical need, ischemic stroke, pre-clinically we find that sGC activity is virtually absent post-stroke. Conversely, a heme-free form of sGC, apo-sGC, was now the predominant isoform suggesting it may be a mechanism-based target in stroke. Indeed, this repurposing hypothesis could be validated experimentally in vivo as specific activators of apo-sGC were directly neuroprotective, reduced infarct size and increased survival. Thus, common mechanism clusters of the diseasome allow direct drug repurposing across previously unrelated disease phenotypes redefining them in a mechanism-based manner. Specifically, our example of repurposing apo-sGC activators for ischemic stroke should be urgently validated clinically as a possible first-in-class neuroprotective therapy.

## Systems based pharmacology: drug repurposing by diseasome

Systems medicine utilizes common genetic origins and co-morbidities to uncover mechanistic links between diseases, which are summarized in the diseasome. Shared pathomechanisms may also allow for drug repurposing within these disease clusters. Here, Schmidt and co-workers show indeed that, based on this principle, a cardio-pulmonary drug can be surprisingly repurposed for a previously not recognised application as a direct neuroprotectant. They find that the cyclic GMP forming soluble guanylate cyclase becomes dysfunctional upon stroke but regains catalytic activity in the presence of specific activator compounds. This new mechanism-based therapy should be urgently validated clinically as a possible first-in-class treatment in stroke.

## Introduction

Drug discovery and development follows a relatively uniform path from mechanistic hypothesis, preclinical disease models to clinical validation. However, in recent years, a string of major drug developments have failed due to lack of efficacy.^1^ One reason for this appears to reside in our current definitions of ‘disease’, i.e., mostly organ-based or by an apparent phenotype or symptom and not by an underlying mechanisms. However, without a validated pathomechanism no mechanism-based drugs can be developed and, therefore, rather surrogate parameters or risk factors are treated instead. Finding a rational approach towards mechanism-based disease definitions may therefore have a tremendous impact on drug discovery and medicine in general.

Using a data-driven approach, disease–disease networks (diseosome) have been constructed in which diseases are linked based on common molecular or regulatory mechanisms,^2^ such as shared genetic associations,^2^ protein interactions^3,4^ or gene–disease interactions.^5^ These diseasomes exhibit local clusters of diseases whose molecular relationships are well understood, but also unexpected clusters of surprisingly heterogeneous diseases.^3^ Such clustering of disease phenotypes is likely due to underlying hidden common pathomechanisms. Importantly, these common mechanism clusters may provide previously unrecognized molecular definitions of these phenotypes and at the same time targets for mechanism-based drug discovery and repurposing.

Here we test the clinical validity of this approach by focusing on one cluster of highly prevalent combinations of vascular, neurological and metabolic disease phenotypes with high unmet medical need. Genetic evidence points to cGMP signaling as being part of its underlying pathomechanism.^5,6^ We then inquire in a non-hypothesis-based manner using disease–disease networks based on common genetic origins, common protein interactions between disease genes, shared disease symptoms and disease co-morbidity for possible drug repurposing of cGMP modulators within this cluster.

## Results

### Human diseasome and protein interactome of sGC in stroke

**Fig. 1:**
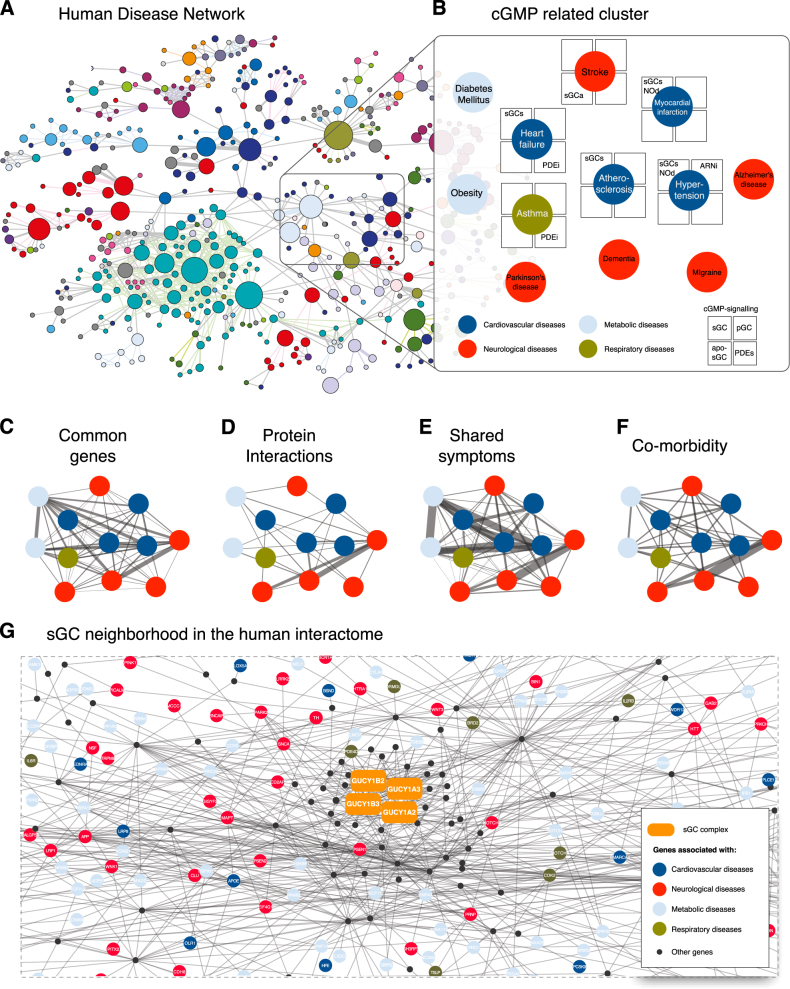
A cGMP-related phenotype cluster within the human diseasome suggests a predominant neurological relevance. **a** shows the human disease network^2^ where nodes represent disease phenotypes that are linked if they share a genetic association. Different colors indicate different organ systems. Within the network a local cluster contains several diseases phenotypes therapeutically amenable to drugs modulating cGMP forming or cGMP metabolizing enzymes. This is magnified in **b** and reveals additional metabolic (blue: DM, diabetes mellitus; OB, obesity), pulmonary (brown: AB, asthma) and neurological (red: ST, stroke; AD, Alzheimer’s disease; MG, migraine; DE, dementia; PD, Parkinson’s disease) linked disease phenotypes. For each disease we included in the squares those drugs that are either in the clinic or in late stage clinical development (sGCa, apo-sGC activators; sGCs, sGC stimulators; NOd, NO donors; ARNi; neprilysin inhibitors; PDEi, phosphodiesterase inhibitors). Panels indicate common associated genes **c**, physical interactions between affected proteins **d**, shared symptoms **e** and elevated comorbidity **f**. When analyzing the interactome of sGC **g**, its subunits (orange) are significantly closer to neurological diseases (red) than random expectation and surprisingly more distant to cardiovascular applications (dark blue) that current clinical practice (Table 2)

The human diseasome provides a framework to pinpoint connections between seemingly distinct diseases.^2^ Built by connecting diseases that share genetic associations, the links in the diseasome suggest common pathophysiology between diseases through pleiotropic genes.^3,7^ Within the diseasome, we focused on a cluster with disease phenotypes of high prevalence and unmet medical need. Figure 1a shows an apparently heterogeneous cluster of several neurological, cardiovascular, metabolic and respiratory diseases. We then systematically characterized the therapeutic potential of the diseases inside this cluster. Five out of twelve phenotypes in this cluster are therapeutically targeted by drugs modulating cGMP-forming or cGMP-metabolizing enzymes, including NO donors in myocardial infarction, sGC stimulators and phosphodiesterase inhibitors (PDEi) in hypertension, and combined angiotensin II type 1 receptor blocker/neprilysin inhibitor (ARNI) in heart failure (see Fig. 1a for details). Taken together, these common treatments suggest a prominent role of cGMP signaling in these disease phenotypes, mostly targeting the NO-responsive sGC.^6^ All drugs currently targeting cGMP clinically—NO donors, sGC stimulators and sGC activators—have almost exclusively cardio-pulmonary indications^8^ such as coronary artery disease,^9^ hypertensive crisis^10^ and pulmonary hypertension,^11^ although some of them are currently being tested in other diseases such as cystic fibrosis (NCT02170025), systemic scleroderma (NCT02283762)^5^ and animal models of kidney diseases.^12^

To systematically validate and quantify the relationships between these diseases, we evaluated the importance of (i) common genes, (ii) protein interactions, (iii) shared symptoms, and (iv) co-morbidity patterns among all disease pairs in this cluster (Fig. 1c–f, see Methods for details). To identify disease pairs that are related to each other, we integrated disease gene, protein interaction, disease symptom and comorbidity information from various sources^3,14,13,^ and we generated updated diseasomes connecting the diseases in the cluster mentioned above. In the updated diseasomes, two diseases were connected based on the existence of common disease-associated genes as well as whether these genes were highly connected with each other in the interactome. To assess the connectedness of the disease genes, we checked the number of interactions connecting the genes from one disease to the genes in the other disease and compared it to the number of interactions one would observe between randomly selected gene pairs (see Methods for details). We also built the co-morbidity and symptoms- based diseasomes in which two diseases were connected by their comorbidity and the symptoms they share, respectively. We found that indeed the diseasomes created using different data sources such as gene overlap, interactions connecting disease genes, symptom similarity and comorbidity complemented each other (Supplementary Table S1) and a subset of the diseases within these diseasomes exhibited substantial relations to one another across all molecular and phenotypic qualities. We observed that, in addition to common genetic origins highlighted by shared gene and interaction based networks, stroke played central role, connecting various metabolic, cardiovascular and neurological diseases in the symptom and co-morbidity based networks (Table S2A–D). Indeed, stroke was either the most central or one of the top connected nodes in these disease–disease networks and had the highest degree centrality when the degree of the diseases was averaged over all networks (Table 1).

**Table 1:** Centrality of diseases in diseasomes, calculated as the degree centrality of each disease within diseasomes and average centrality across four diseasomes

Disease	Gene network degree	Interaction network degree	Symptom network degree	Comorbidity network degree	Average degree
Stroke	10	10	2	7	7.25
Alzheimer disease	6	9	1	5	5.25
Dementia	6	10	3	0	4.75
Atherosclerosis	3	6	0	8	4.25
Asthma	3	7	1	5	4
Diabetes mellitus^a^	6	10	0	0	4
Parkinson disease	3	7	2	4	4
Heart Failure	1	4	2	7	3.5
Migraine Disorders	1	7	0	5	3.25
Myocardial Infarction	4	7	2	0	3.25
Hypertension	1	3	1	7	3
Obesity	2	4	0	6	3

^a^ Diabetes mellitus type 2

To elucidate whether sGC might be related to stroke or other disease phenotypes within this cluster, we determined the interactome-based proximity^14^ of the proteins within the sGC complex to the proteins associated with major common diseases. Surprisingly, the network-based analysis identified sGC not only as a promising target in non-smooth muscle but suggested that its main mechanism-based indication should be in neurological diseases. Of these, vision disorders, ataxia and, again, stroke stood out. Previously, only very few observations had pointed in that direction without being clinically exploited, neither in ataxia nor retinitis pigmentosa.^15^ All three known subunits of heterodimeric sGC (alpha-1, alpha-2, and beta-1) were significantly close to genes known to be involved in stroke (Table 2), leading to a joint z-score of −2.70 but only one report had linked a knock-out of the sGC alpha-1 subunit to impaired vascular smooth muscle reactivity and larger ischemic infarcts.^7^ However, the finding of a phenotype in ischemic stroke by knocking out a vasodilatory enzyme such as sGC is not necessarily a lead. In fact, knocking out any vasodilatory gene or overexpressing a vasoconstrictive gene will have that effect. Moreover, mice with a deletion of either the sGCalpha-1 or beta-1 gene are hypertensive^16^ and any hypertensive mouse will show a higher susceptibility to a worsened outcome in experimental stroke; however, at the same time we know that all tested anti-hypertensives were unsuccessful or dangerous in stroke therapy.^17^ Finally, NO, the specific stimulator of sGC, is toxic in stroke^18^ precluding its use and sGC as a therapeutic target.

**Table 2:** Interactome-based proximity from sGC subunits to various disease phenotypes

sGC subunit^a^	Stroke	Disease	Z^b^	*p*-value^c^
All		Vision disorders	−3.405	0.00033
		Varicose veins	−3.135	0.00086
		Esophageal disease	−3.001	0.00134
		Spinocerebellar ataxias	−2.995	0.00137
		Asthma	−2.787	0.00266
		Head and neck neoplasms	−2.725	0.00321
	*	Stroke	−2.698	0.00348
		Retinitis pigmentosa	−2.279	0.01132
		Diabetes mellitus type 2	−2.122	0.01691
α-1		Vision disorders	−3.711	0.00010
		Varicose veins	−3.239	0.00060
		Esophageal disease	−2.959	0.00154
		Spinocerebellar ataxias	−2.741	0.00307
		Head and neck neoplasms	−2.529	0.00572
		Asthma	−2.494	0.00631
	*	Stroke	−2.288	0.01106
		Retinitis pigmentosa	−1.923	0.02727
		Diabetes mellitus type 2	−1.778	0.037678
α-2^d^		Vision disorders	−2.383	0.00859
		Spinocerebellar ataxias	−2.356	0.00923
	*	Stroke	−2.289	0.01105
		Varicose veins	−2.240	0.01255
		Esophageal disease	−2.238	0.01263
		Asthma	−2.217	0.01330
		Head and neck neoplasms	−2.127	0.01672
		Retinitis pigmentosa	−2.108	0.01751
		X-linked mental retardation	−2.080	0.01874
β-1^e^		Vision disorders	−2.387	0.00848
	*	Stroke	−2.339	0,00966
		Asthma	−2.291	0.01097
		Varicose veins	−2.270	0.01162
		Spinocerebellar ataxias	−2.266	0.01172
		Head and neck neoplasms	−2.225	0.01302
		Esophageal disease	−2.199	0.01394
		Retinitis pigmentosa	−2.041	0.02062
		Diabetes mellitus type 2	−1.931	0.02671

^a^ sGC is an heterodimer requiring both one α and one β subunit

^b^ For each protein and diseases, the proximity quantifies the significance of closeness of the set of proteins to the disease-genes, producing negative z-scores for protein–disease pairs that are closer in the interactome compared to random expectation

^c^ The *p*-value was calculated using the proximity score (z), corresponding to the probability that a randomly selected protein having the same degree in the interactome as the protein of interest would be at least as close to the disease genes than the observed distance in the interactome

^d^ For stroke (annotated as cerebrovascular disease in MeSH), α_2_-subunit, which is predominantly expressed in neurons has the third highest score

^e^ The active β_1_ subunit has the second highest score

Nevertheless, since ischemic stroke is the second cause of death in industrialized countries and the first leading cause of disability and because of our systems-level support for this neurological^11^ indication, we investigated this possible association and sGC drug repurposing opportunity. Moreover, despite this high medical and socio-economical need, no neuroprotective drug is commercially available and close to 85% of patients have no therapeutic option.

### sGC activity and protein levels in ischemic stroke

**Fig. 2:**
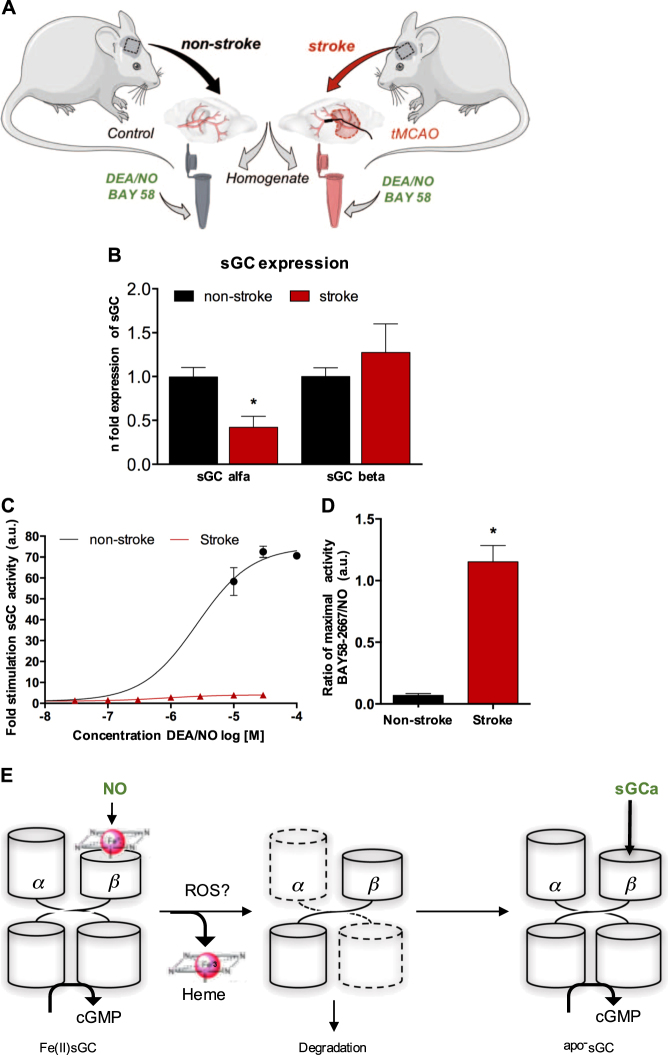
Decreased sGC expression and activity upon ischemic stroke. We tested the primary role of sGC in stroke in a mouse model of transient middle cerebral artery occlusion (tMCAO). **a** Collection of the right brain hemispheres 24 h after tMCAO (stroke) or from control animals (non-stroke) and subsequent cryo-homogenization in sGC assay buffer. Subsequently, the expression of sGCα and sGCβ subunits were measured by Western blot **b** sGC activity as cGMP formation **c**, **d**, in the absence or presence of the NO donor, DEA/NO, which stimulates the Fe(II) heme-containing native sGC, or apo-sGC activator (sGCa), BAY58-2667, which activates the heme-free apo-sGC (see Methods). sGCα expression (*n* = 5, *p* < 0,05), in **b** and NO-Fe(II)sGC activity (*n* = 5) were significantly reduced after stroke. **c**, **d** This loss of NO-Fe(II)sGC activity resulted in a near complete shift in the cGMP response being post-stroke largely dependent on apo-sGC activation (*n* = 3, *p* < 0,05). **e** Loss of heme in the NO-responsive Fe(II)sGC upon stroke, which may possibly involve ROS formation (19). The resulting heme-free apo-sGC is prone to degradation of its α subunit.^50^ cGMP formation can be partially recovered by apo-sGC activation, which binds to the empty heme pocket of apo-sGC opening up a mechanism-based therapeutic option

We examined whether upon ischemic stroke, any abnormalities in sGC become apparent that could suggest sGC as a mechanism-based target. In order to adhere to the quality criteria of pre-clinical stroke research (Stroke Treatment Academic Industry Roundtable (STAIR)) recommendations, we used the most accepted model of transient middle cerebral artery occlusion (tMCAO) in mice of different age and gender (Fig. 2a). We analyzed the acute phase of ischemic stroke, because many consequences of stroke (e.g., infarct volume, blood-brain barrier disruption, neurological deficits, cell death) can already be observed at 24 h post-ischemia. We find that 24 h after onset of tMCAO, sGC alpha but not beta protein levels were significantly reduced in homogenates of stroked vs. non-stroked mouse brains (Fig. 2b). sGC is broadly distributed in different brain structures such us telencephalon, thalamus, hypothalamus being expressed mainly in neurons, glial and endothelial cells of the brain microvasculature.^19,20^ These findings are also conserved in human brain tissue based on The Human Protein Atlas.

Surprisingly, the remaining sGC protein was not responsive to NO (Fig. 2c), arguing against sGC being a target. This observation and selective reduction of sGC alpha was reminiscent of an oxidative stress-mediated mechanism^21^ favouring the appearance of a different isozyme, heme-free apo-sGC, which is unresponsive to NO and prone to degradation.^22^ Thus rather than sGC (as suggested by sGC alpha-1 knock out^7^) but apo-sGC appeared to be a possible pathomechanism and pharmacological target. Indeed, specific apo-sGC activator compounds are available, which bind to the empty heme pocket of apo-sGC^23^ to elevate cGMP under disease conditions.^24^

In agreement with a mechanism-based role of apo-sGC, post-stroke apo-sGC activation was fully preserved and even slightly increased. The net resulted of these stroke-induced changes was therefore a dramatic shift of the sGC/apo-sGC ratio resulting in complete dependence of cerebral cGMP formation on apo-sGC (Fig. 2d, e).

### Infarct size, blood brain barrier disruption and survival upon post-stroke apo-sGC activation

Having established a mechanism-based appearance of apo-sGC upon stroke, we next wanted to examine whether post-stroke pharmacological targeting of apo-sGC by an apo-sGC activator may have the potential of a mechanism-based treatment, i.e. repairing the loss of physiological NO-induced cGMP formation by generating cGMP through apo-sGC. Stroke pathophysiology leads to an increase in blood–brain barrier (BBB) permeability allowing compounds crossing and targeting neuronal signaling pathways.

**Fig. 3:**
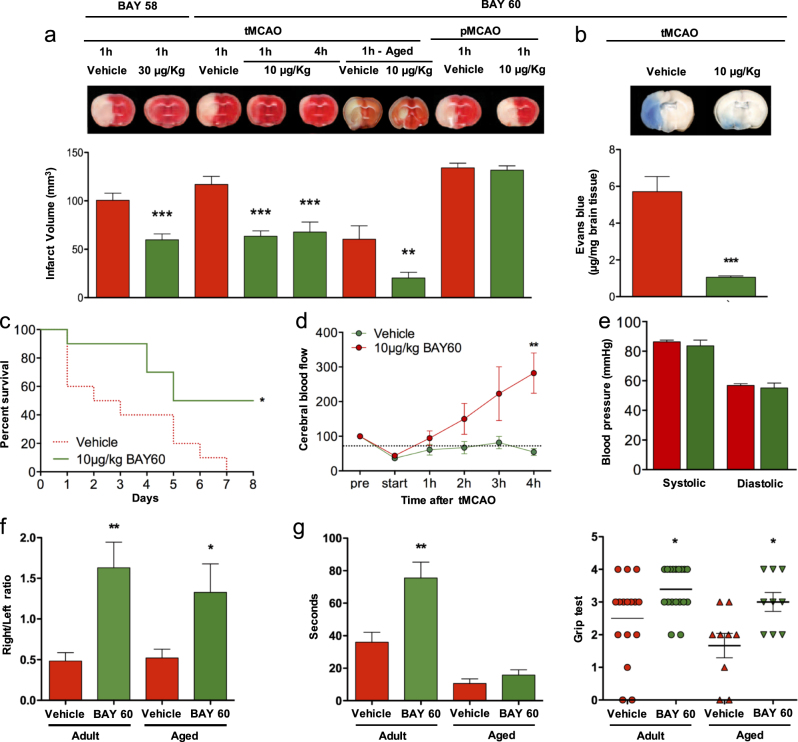
Post-stroke treatment with either of two apo-sGC activators reduces infarct size, prevents blood-brain barrier disruption, improves two of three neurological outcomes and increases survival in a reperfusion-dependent manner. **a** 24 h after transient (with reperfusion) but not permanent (without reperfusion) occlusion of the middle cerebral artery (tMCAO and pMCAO, resp.) infarct size is reduced in mice treated with BAY58-2667 (30 μg/kg) 1 h post-stroke (*n* = 10, *p* < 0,001) and BAY60-2770 (10 μg/kg) 1 h (*n* = 19, *p* < 0,001) and 4 h (*n* = 10, *p* < 0,001). Infarct volume was also significantly reduced in middle-aged animals treated with BAY60-2770 (10 μg/kg) 1 h post-stroke (*n* = 8, *p* < 0,01). **b** Mechanistically, BAY60-2770 treatment (5 and 10 μg/kg) reduced blood- brain barrier leakage in a dose dependent manner (*n* = 10, *p* < 0,0001). **c** Apo-sGC activation treatment increased survival as soon as after day 1, which persisted until day 8, when 100% of control mice had died (*n* = 10, *p* < 0.05). **d** Post-stroke cerebral blood flow was increased in response to BAY60-2770 (*n* = 5, *p* < 0.05) while **e** systemic blood pressure was not modified at the chosen dose (10 μg/kg) (*n* = 4). **f**–**h** With respect to the neurological outcome of BAY60-2770 treatment in surviving mice, the elevated body swing test indicated a significant increase for the right swing number/total swing number ratio both in adult (*n* = 19, *p* < 0.01; panel **f**) and middle-aged mice (*n* = 8, *p* < 0.05; panel f) while significantly improved motor outcome after four limb hanging test was only seen in the adult model (*n* = 19, *p* < 0.05, panel **g**). Similarly, the grip-test was significantly improved in both mice models (*n* = 19, *n* = 8, *p* < 0.05, panel **h**)

Since sGC activators (sGCa) can dilate blood vessels and have hypotensive side-effects, hemodynamically ineffective doses were chosen for this study. We subjected 8-week-old male and female C57Bl/6 mice to tMCAO and treated with either of two apo-sGC activators, BAY58-2667 or BAY60-2770, at two different doses (30 µg/kg and 10 µg/kg of body weight) respectively, either 1 h or 4 h post-stroke induction. To exclude sex-specific effects, both 1-year-old female and male C57Bl/6 mice were also subjected to tMCAO and treated with BAY60-2770 (10 µg/kg) 1 h post-stroke. To asses also an alternative stroke model, 8-week-old male and female C57Bl/6 mice were subjected to permanent occlusion of the MCA (pMCAO) and treated with BAY60-2770 (10 µg/kg) 1 h post-stroke. In all cases, infarct volumes were assessed after 24 h by staining brain sections with 2,3,5-triphenyltetrazolium chloride (TTC). Consistent with a pathomechanistic role of apo-sGC in stroke, the BAY60-2770- or 58-2667-treated mice developed significantly smaller brain infarctions on day 1 after tMCAO compared to controls. No effect was observed in pMCAO suggesting a reperfusion-dependent effect (Fig. 3a) further arguing against any vasodilatory component of this effect.

To further analyze the pharmacodynamics of this effects, we focused on BAY 60-2770, which at the lower dose consistently had a slightly higher efficacy. Mechanistically, cGMP is suggested to be involved in the regulation of vascular permeability.^25^ Therefore, we analyzed the integrity of the blood-brain barrier after ischemic stroke measuring the extravasation of the vascular tracer Evans Blue into the brain parenchyma. Indeed, BAY60-2770 treatment (10 mg/kg) significantly reduced BBB disruption in a dose-dependent manner (Fig. 3b).

In addition to infarct size reduction and BBB stabilization, survival is an even more relevant outcome parameter. We therefore also determined mortality over a time of seven days after stroke. At day four, 6 of 10 animals in the vehicle group had died, but just one in the BAY60-2770 treated group. Seven days after stroke, 9 of 10 mice had died in the control group; in the BAY60-2770 treated group, only 5 out of 10 (Fig. 3c).

### Cerebral blood-flow vs. systemic blood pressure upon apo-sGC activation

As a point of caution, apo-sGC activators have been shown to be directly vasoactive.^22^ Vasoactive drugs (or knock-out models^7^) bear the risk of dilating healthy blood vessels more efficiently than those blood vessels affected by the disease condition.^26^ In consequence, a paradoxical “steal phenomenon” may be observed shunting blood from diseased tissue to healthy vascular beds. Moreover, an additional drop in systemic blood pressure may lower perfusion pressure^15^ and affect ischemic reperfusion. We therefore studied apo-sGC activator treatment over time (during 4 h) to compare the effects on systemic blood pressure vs. relative regional cerebral blood flow (rCBF) in the territory of the right middle cerebral artery as measured by Laser Doppler Flowmetry.^27^ Importantly, no differences in baseline rCBF (100% before ischemia) nor immediately after ischemia (insertion of the occluding filament) were observed, indicating comparable procedural conditions in both groups. Directly after removal of the filament, one group of mice was injected with 10 µg/kg BAY60-2770. From that point on, we observed a higher rCBF in the treated mice compared to the control group. In the control group the rCBF rose within the next 2 h after reperfusion (3 h after MCAO) reaching a maximum of about 80% of the baseline level followed by a recurrent drop of rCBF at 3 h after reperfusion. In contrast, in the BAY60-2770 group, rCBF increased steadily until 4 h after ischemia. Of note, rCBF strikingly increased and reached a level that was nearly threefold higher than baseline (Fig. 3d). No significant influence of 10 µg/kg BAY60-2770 on systemic blood pressure was observed (Fig. 3e).

### Improved neurological outcome upon post-stroke apo-sGC activation

Clinically, smaller infarct and survival would be important, but so would quality of life and/or neurological function in survivors. Thus, we additionally assessed three independent neuro-functional parameters both in the adult and middle-aged mouse model treated 1 h post-stroke, i.e., the elevated body swing test (Fig. 3f), the four-limb hanging wire test (Fig. 3g), and the Grip test (Fig. 3h). In the middle-aged model, only the elevated body swing test and Grip test showed significant improvement, while in the adult model, importantly, all three neuro-functional outcome parameters were significantly improved. Thus, activating post-stroke elevated apo-sGC reduced infarct volume, increased survival, and in surviving animals protected neuro-motor function.

### Effects of apo-sGC activation on microglia and early inflammation

Upon stroke, both interleukin-1β (IL-1β)^28^ and tumor necrosis factor-α (TNF-α),^29^ are induced early^30^ in microglia and infiltrating macrophages contributing to neurodegeneration.^31–33^ To assess whether these events are also related to apo-sGC, we first assessed microglia activation and infiltrating macrophages in stroked animals with and without treatment with the apo-sGC activator BAY 60-2770 (10 µg/kg). Indeed, 24 h post-treatment microglia activation and infiltration of macrophages was significantly reduced by BAY 60-2770 compared with non-treated controls (SI Fig. S1). Consistent with this, expression of IL-1β and TNF-α were significantly reduced by BAY 60-2770 treatment 4 h post-stroke (SI Fig. S2A-B). These data corroborate the key role of apo-sGC and its pharmacological targeting in stroke.

### Direct neuroprotection via cGMP-dependent protein kinase (PKG)/p-CREB

**Fig. 4:**
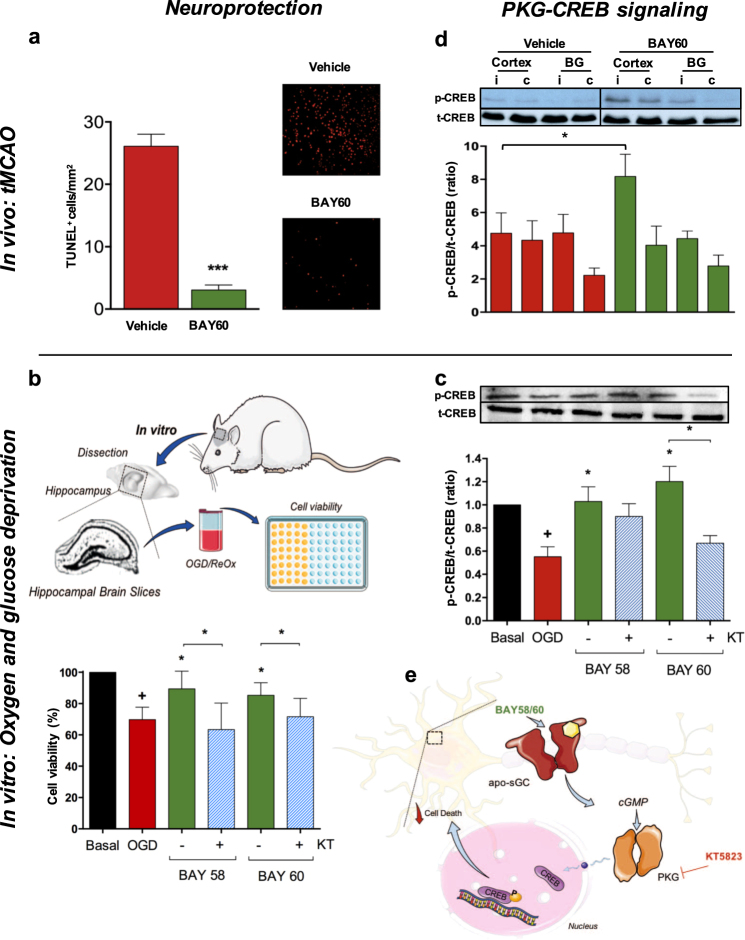
Both in vitro and in vivo sGC activation is directly neuroprotective in a cGMP-PKG-CREB dependent manner. **a** Improved outcome in treated mice (see Fig. 3) was associated with decreased cortical neuronal apoptosis (*n* = 4, *p* < 0.001) suggesting a novel neuroprotective mechanism. **b** shows an in vitro ischemia model free of vascular and blood-brain-barrier components. After dissection, hippocampal brain slices were subjected to oxygen and glucose deprivation (OGD)/re-oxygenation (ReOx) in the absence or presence of the sGC activators, BAY58-2667 or BAY60-2770, with and without the cGMP-dependent protein kinase inhibitor, KT5823.^61^ Both BAY58-2667 (*n* = 7, *p* < 0.05) or BAY60-2667 (*n* = 13, *p* < 0.05) increased cell viability after OGD/Re-Ox. Adding the PKG-inhibitor KT5823 reversed this effect for both BAY58-2667 (*n* = 8, *p* < 0.05) and BAY60-2770 (*n* = 7, *p* < 0.05). **c** BAY60-2770 treatment increased the ratio of the PKG substrate *p*-CREB/t-CREB upon OGD/ReOx (*n* = 8, *p* < 0.05), an effect was completely prevented in the presence of the PKG-inhibitor KT5823 (*n* = 4, *p* < 0.05), suggesting a neuroprotective link. **d** Similarly, p-CREB/t-CREB ratio was significantly increased in vivo 24 h post-stroke (*n* = 4, *p* < 0.05). **e** Schematic representation of the suggested neuroprotective signaling events: BAY58-2667/60-2770 specifically activates the predominant post-stroke form of sGC (heme-free apo-sGC) and thereby recovers lost sGC-dependent cGMP formation, which leads to CREB phosphorylation and direct neuroprotection

Since the absence of hypotension and dependence on reperfusion suggested that apo-sGC activators were acting via a previously unrecognized direct neuroprotective effect in agreement with the diseasome guided primary neuronal disease association, we investigated their direct effects on hypoxia-induced neuronal apoptosis, a well-established mechanism of tissue damage in ischemic stroke.^34^ Indeed, 24 h after stroke onset immunolabeling of TUNEL and NeuN revealed widespread apoptosis in the vehicle treated mice. In contrast, the number of apoptotic cells in the BAY60-2770 treated animals was significantly reduced (Fig. 4a).

Moreover, we studied an additional in vitro model free of functional vascular components, i.e., hippocampal slices that maintain their morphology and properties of synaptic function similar to those under in vivo conditions. We simulated an ischemic stroke and reperfusion scenario by exposing the slices to a 15 min period of oxygen and glucose deprivation (OGD), followed by a 2 h period of re-oxygenation (ReOx) in the absence or presence of the sGC activators, BAY58-2667 (100 nM) or BAY60-2770 (100 nM). In some experiments, the inhibitor of cGMP-dependent protein kinase (PKG), KT5823 (60 nM), was added to either of the apo-sGC activators. When determining hippocampal cell viability by the MTT assay, both apo-sGC activators were directly neuroprotective upon OGD. This protection was completely lost when the PKG inhibitor KT5823 was present (Fig. 4b) suggesting that both apo-sGC activators exerted their protection by elevating cGMP and PKG activity.

Since the transcription factor CREB has been described as a key mechanism of neuronal growth and survival,^35^ we considered its involvement as a possible phosphorylation substrate of PKG.^36^ Indeed, the apo-sGC activator BAY60-2770 significantly increased CREB phosphorylation, an effect that was completely prevented in presence of the PKG inhibitor, KT5823 (Fig. 4c). Consistent with this, CREB phosphorylation was significantly increased in tMCAO mice treated with BAY60-2770 (Fig. 4d) correlating with its anti-apoptotic effects in vivo. Taken together, our data suggest that post-stroke apo-sGC but not sGC is a mechanism-based target and its activation is directly neuroprotective via PKG-CREB dependent signaling (Fig. 4e).

## Discussion

Here we apply a new approach to disease definition, target identification and validation. We also provide a top-down drug repurposing strategy and experimental confirmation of drug repurposing based on the human diseasomes generated by not only common genetic origins but also shared protein interactions, symptom similarity and comorbidity.

Shared gene associations can offer mechanistic insights into common pathophysiology of seemingly distinct diseases. The human diseasomes leverages such genetic and clinical commonalities among diseases, giving rise to key applications from characterization of disease comorbidities to drug repurposing.^3^ Unlike previous studies that rely mainly on common genetic origins, we exploit large-scale disease and interaction data for hypothesis generation within a cluster of apparently mechanistically related cerebro-cardio-metabolic disease phenotypes with high prevalence and unmet medical needs. Genetic^6,7^ and pharmacological^5,37,38^ evidence had suggested that these are associated with and defined by (an impairment of) cGMP signaling. Therefore we focused our non-hypothesis-based approach on the cGMP generating soluble guanylate cyclase, currently clinically targeted mainly for cardiovascular indications.

Previous studies suggest increased cGMP levels may interact in modulating hypoxic stimulation of erythropoietin (EPO) production.^39,40^ Likewise, post-stroke treatment with erythropoietin showed neuroprotective effects in cerebral ischemia^41^ by reducing neuroinflammation^42^ and promoting synaptic plasticity.^43^ Therefore, pharmacological treatment post-stroke using sGC activators may also stimulate erythropoietin production leading to a secondary neuroprotective pathway. However, we believe that a mechanism-based upstream effector such as sGC having multiple downstream effectors, such as EPO, direct neuroprotection, and cytokines, has a higher chance of being therapeutically relevant. This may also explain why EPO-only therapeutic approaches in stroke failed eventually in the clinic.^44^

We combine in silico, in vitro, and in vivo methods and surprisingly find previously not recognized neurological disease phenotypes as the most promising indication for this target. Moreover, when selecting out of these the indication stroke, we do not find NO-sensitive sGC but a pharmacologically entirely different isoform, apo-sGC, to be the target. Indeed, using a set of apo-sGC specific activator compounds, we observe direct neuroprotection independent of vascular effects.

Functionally, this led to reduced infarct size, reduced mortality and improved neurological outcome in surviving animals, independent of sex and age. Thus apo-sGC activators should be urgently examined for re-purposing for the high unmet medical need indication, stroke, and possibly other neurological disease phenotypes such as vision disorders, spinocerebellar ataxias and retinitis pigmentosa.^15^ apo-sGC activator compounds, currently in phase II clinical development for heart failure,^45^ a chronic cardiovascular indication, should be examined in phase IIb/III acute studies in low non-hypotensive doses in ischemic stroke. Because of no apparent bleeding risk, therapy may be started earlier, in the ambulance as soon as a stroke is suspected in a patient; it may also have a wider time-window than current t-PA and may be extended to neuroprotection in hemorrhagic stroke for which no pharmacotherapy is available. This lack of definitive clinical proof-of-concept is of course still the major limitation of our present combined in silico and pre-clinical findings. Mechanistically, PKG-CREB-dependent signaling seemed to mediate these effects.

On a more general scale, our sequence of in silico diseasomes cluster-based target identification and subsequent in vivo/in vitro validation may represent an innovative, non-hypothesis driven approach to drug discovery and repurposing for other unmet medical needs when classical hypothesis-driven discovery approaches have resulted in unsatisfactory clinical efficacy. It is possible to repurpose within a common mechanism cluster one target—in our case sGC—from one organ system (cardiovascular) to another (neurological). However, genetic definition of this target, i.e., sGC, would have been insufficient as surprisingly a protein isoform, apo-sGC and specific activator compounds, provided the key discovery. It is unclear whether apo-sGC was derived from sGC (by heme loss) or de novo from incomplete heme insertion.^46^

Common molecular mechanisms, such as cGMPopathies, defining at least partially the present cluster, may be a more precise and molecular denominator of disease rather than describing an organ- or symptom-based phenotype and could lead to an entirely new definition of disease.^47,48^ Basing a disease on an underlying molecular mechanism will by definition provide a translational handle, both with respect to diagnostics and mechanism-based therapeutics including, as we show here, rapid clinical repurposing opportunities.

## Methods

### Study design

All stroke experiments were performed in accordance with the recently published ARRIVE guidelines (http://www.nc3rs.org/ARRIVE). An independent person not involved in data acquisition and analysis randomly assigned animals to the operators. We performed surgery and evaluation of all read-out parameters while being blinded to the experimental groups. Animals were excluded from end-point analyses (exclusion criteria) if death occurred within 24 h after MCAO, if subarachnoidal hemorrhage (SAH) or intracerebral hemorrhage (ICH) occurred (as macroscopically assessed during brain sampling), or if, 60 min after tMCAO, Bederson scores were = 0. Of the 242 mice subjected to MCAO, 38 mice (15.7%) met at least one of these exclusion criteria after randomization and were withdrawn from the study resulting in 204 included mice. The drop-out rates was 22 mice in the vehicle group vs. 16 mice in the BAY60-2770 treated group. The study design was defined based on a power animal calculation analysis^49^ for all groups involved in the study considering different compounds (BAY58-2667 and BAY60-2770), doses (30 µg/kg, 10 µg/kg), treatment time-points (1 h and 4 h post-stroke), age of the animals (adults and middle-aged) based on mean infarct size, standard deviation a and number of animals used always comparing vehicle and treatment administration (Table S3). The study was conducted in accordance with institutional guidelines (University of Würzburg, Germany) for the use of experimental animals, and the protocols were approved by governmental authorities (Regierung von Unterfranken, Würzburg, Germany).

### Human interactome analysis

We used an integrated human interactome containing 141,150 experimentally documented physical interactions between 13,329 proteins, as curated by ref. ^3^ From the same study, we used 299 diseases defined by MeSH classification and their associated genes that were curated from OMIM and GWAS databases to identify disease modules within the interactome. To remove potential redundancy among diseases, we calculated the Jaccard Index between every pair of diseases using the genes associated to them and filtered the disease terms that had a Jaccard Index higher than 0.5 to a disease in the data set, yielding 132 diseases. For each of the sGC proteins, guanylate cyclase soluble subunit beta-1 (*GUCY1B3*), guanylate cyclase soluble subunit alpha-2 (*GUCY1A2*) and subunit alpha-3 (*GUCY1A3*), we calculated the minimum shortest path length to known disease genes for each of the 132 diseases. As control, we further computed the minimum shortest path lengths from the sGCα proteins to 1000 randomly chosen genes sets containing the same number of genes as the respective disease. From the mean and standard deviation of these random controls we then calculated a *z*-score, *z* = (*d*_observed_−<*d*_random_>)/σ_random_, to quantify the significance of the closeness of the sGCα proteins to a given disease. We converted *z*-scores to *P*-values and then adjusted the *P*-values using the Benjamini–Hochberg multiple hypothesis testing correction method. We set the alpha value for FDR to 25% and considered any disease with an FDR less than or equal to this value as significantly close to the sGCα protein.

### Disease–disease relationships

The four different relationships among the diseases in the cluster depicted in Fig. 1c–f were obtained as follows: (i) We determined the number of shared genes (Fig. 1c) from gene-disease association data combined from ref. ^3^ to ref. ^50^ (ii) We computed the number of physical protein interactions connecting the gene products of the two respective diseases using interactome data from.^3^ For every disease pair, we first checked the number of interactions connecting the genes from one disease to the genes in the other disease (see the Supplemenary Material for the pseudocode for this procedure). We then compared the number of observed interactions between disease genes to the number of interactions one would observe between randomly selected gene pairs and used the normalized score (z-score) to decide whether the disease genes were more connected to each other compared to random pairs of genes. The expected distribution of the number of interacting gene pairs was generated using 1000 randomly chosen pairs of gene sets containing the same number of genes as the two diseases in consideration. The *z*-score was calculated using the mean and standard deviation of this distribution. Links in Fig. 1d indicate a significant number of interacting proteins. (iii) We extracted disease pairs with high symptom similarity^4^ (Fig. 1e). Only the symptoms that had strong support (TF-IDF score > 3.5) in the original data set were considered in the analysis. (iv) Elevated co-morbidities calculated from disease pairs reported in insurance claims (Fig. 1f) were taken from ref. ^51^ The ICD9 codes provided in the original data set were converted to MeSH identifiers using the mapping provided by Disease Ontology and only the diseases that could be unambiguously mapped were included in the analysis. The pairwise disease–disease relationship scores corresponding to the calculated gene and interaction sharing, symptom similarity and comorbidity are available in Tables S2A-D.

### Centrality analysis of diseases in diseasomes

To prioritize the diseases, we generated four diseasomes based on the disease–disease relationship scores above. We kept only the links that correspond to non-spurious relationships between diseases by including disease pairs that shared at least one gene (*n* > 0); had positive shared protein interaction score (z > 0); showed symptom similarity (Jaccard index > 0.5); and are known to be comorbid (relative risk > 1) in the four diseasomes, respectively. We then calculated degree centrality of each disease within these diseasomes and averaged the centrality values as the final centrality value of the disease across diseasomes (Table 1). To ensure that the incompleteness of the interaction data did not have a significant effect on the ranking of the diseases, we also checked the centrality of the diseases without using the interactome-based diseasome and found that stroke remained as the most central disease across the diseasomes (Table S4). The coverage and average degree of the individual diseasomes are given in Table S1.

### sGC activity

The determination of sGC activity was performed in homogenates of mouse stroked ipsilateral cortex and basal ganglia vs. non-stroke cortex and basal ganglia, as previously described.^23^ Briefly, crude brain homogenates of stroked and non-stroked C57Bl/6 mice were measured as the formation of cGMP at 37 C during 10 min in a total incubation volume of 100 μl containing 50 mM triethanolamine–HCl (pH 7.4, Sigma), 3 mM MgCl, 3 mM glutathione (Carl Roth), 1 mM 3-isobutyl-1-methylxanthine (IBMX, Enzo LifeSciences), 100 mM zaprinast (Enzo LifeSciences), 5 mM creatine phosphate (CalBiochem), 0.25 mg/ml creatine kinase (CalBiochem), and 500 mM GTP. The reaction was started by simultaneous addition of the crude brain homogenates and either DEA/NO (Enzo LifeSciences) or BAY58-2667 (Adipogene), respectively. After incubation of each sample (*n* = 3 each per group) for 10 min the reaction was stopped by boiling for 10 min at 95 °C. Thereafter, the amount of cGMP was subsequently determined by an enzyme immunoassay (ENZO cGMP EIA kit) using different sample dilutions in the linear range.

### In vivo MCAO ischemia model

C57BL/6 mice were subjected to middle cerebral artery occlusion (MCAO) followed by 24 h of reperfusion. Focal cerebral ischemia was induced by 60 min transient middle cerebral artery occlusion (tMCAO) as described in refs. ^52–54^ Mice were anesthetized with 2.5% isoflurane (Abbott) in a 70% N_2_O/30% O_2_ mixture. Core body temperature was maintained at 37 °C throughout surgery by using a feedback-controlled heating device. Following a midline skin incision in the neck, the proximal common carotid artery and the external carotid artery were ligated and a standardized silicon rubber-coated 6.0 nylon monofilament (6021; Doccol) was inserted and advanced via the right internal carotid artery to occlude the origin of the right MCA. The intraluminal suture was left in situ for 60 min. Then animals were re-anesthetized and the occluding monofilament was withdrawn to allow for reperfusion. For permanent MCAO (pMCAO) the occluding filament was left in situ until sacrificing the animals.^54^ Operation time per animal did not exceed 10 min.

### Treatment with apo-sGC activators

BAY60-2270 and BAY 58-2667 were dissolved in a mixture of Transcutol/Cremophor/water in a ratio of 10/20/70.^55^ BAY 58-2667 (30 µg/kg), BAY60-2270 (5 µg/kg or 10 µg/kg) or vehicle (Transcutol/Cremophor/water in a ratio of 10/20/70) was injected i.v. at the time of reperfusion (immediately after removal of the filament) except in one group where BAY 60-2270 was injected 3 h after removal of the filament that means 4 h after induction of tMCAO.

### Determination of blood-brain-barrier leakage and brain edema

To determine blood–brain–barrier leakage 100 µl of 2% Evan’s Blue tracer (Sigma Aldrich) diluted in 0.9% NaCl was i.v. injected 1 h after the induction of tMCAO. After 24 h mice were sacrificed and brains were quickly removed and cut in 2 mm thick coronal sections using a mouse brain slice matrix (Harvard Apparatus). Brain slices were fixed in 4% PFA at 4 °C for 2 h in the dark. Then, brain slices were cut into small pieces using a scalpel and then transferred into Eppendorf tubes. 500 µl Formamid was added to each tube and incubated for 24 h at 50 °C in the dark. Tubes were centrifuged for 20 min at 16,000 g and 50 µl of the supernatant was transferred to a 96 well plate. Fluorescence intensity was determined in duplicates by a microplate fluorescence reader (Fluorosan Ascent, Thermo Scientific) with an excitation at 620 nm and emission at 680 nm. The concentration for each sample was calculated from a standard curve using linear regression analysis.

### Invasive hemodynamics

For the assessment of blood pressure mice were anesthetized with 2.0% isoflurane and catheterized via the right carotid artery with a high-fidelity 1.4 F Millar microtip catheter (Milar Instruments), as described in ref. ^56^ Hemodynamic data were digitized via a MacLab system (AD Instruments) connected to an Apple G4 PowerPC computer and analyzed.

### Laser Doppler flowmetry

Laser Doppler flowmetry (Moor Instruments) was used to monitor relative regional cerebral blood flow (rCBF) in the right MCA area.^57^ For this procedure, a small incision was made in the skin overlying the temporal muscle, and a 0.7 mm flexible Laser Doppler probe (model P10) was positioned perpendicular to the superior portion of the temporal bone (6 mm lateral and 2 mm posterior from bregma). This position corresponds to the core of the ischemic territory. rCBF was measured serially at baseline (before ischemia), immediately after insertion of the occluding filament (ischemia), immediately after removal of the occluding filament (reperfusion) and again 1 h, 2 h, and 3 h after reperfusion.

### Determination of infarct size

After sacrificing the mice, brains were quickly removed and cut in three 2-mm thick coronal sections using a mouse brain slice matrix (Harvard Apparatus). The slices were stained for 10 min at 37 °C with 2% 2,3,5-triphenyltetrazolium chloride (TTC; Sigma-Aldrich) in PBS to visualize the infarctions.^58^ Indirect, i.e., corrected for brain edema, infarct volumes were calculated by volumetry (ImageJ software, National Institutes of Health, USA) according to the following equation: *V*_indirect_ (mm^3^) = *V*_infarct_×(1−(*V*_ih_−*V*_ch_)/*V*_ch_), where the term (*V*_ih_−*V*_ch_) represents the volume difference between the ischemic hemisphere and the control hemisphere and (*V*_ih_−*V*_ch_)/*V*_ch_ expresses this difference as a percentage of the control hemisphere.

### Assessment of neuro-functional outcomes

Three different neuro-motor functioning tests were assessed in all mice groups (male, female, middle-aged) treated 1 h post-ischemia. For the grip test^59^ the mouse was placed midway on a string between two supports and rated as follows: 0, falls off; 1, hangs on to string by one or both fore paws; 2, as for 1, and attempts to climb on to string; 3, hangs on to string by one or both fore paws plus one or both hind paws; 4, hangs on to string by fore and hind paws plus tail wrapped around string; 5, escape (to the supports). For the elevated body swing test the mice was held ~1 cm from the base of its tail. Then, it was elevated above the surface in the vertical axis. A swing was considered whenever the animal moved its head out of the vertical axis to either the left or the right side (> 10°). To evaluate limb strength, the four-paw wire hanging test was performed. The mouse was placed on the center of the wire with a diameter of 8 cm and then the wire was slowly inverted and placed at 40 cm above a paper towel bedding. The time until the mouse fell from the wire was recorded, and the maximum time was set to 120 s.

### Staining of activated microglia/macrophages

Cryo-embedded slices were fixed in 4% PFA in PBS. Blocking of epitopes was achieved by pre-treatment with 5% bovine serum albumin (BSA) in PBS for 45 min to prevent unspecific binding. Rat anti-mouse CD11b (microglia/macrophages; MCA711, AbD Serotec) at a dilution of 1:100 in PBS containing 1% BSA was added overnight at 4 °C. Afterwards, slides were incubated with a biotinylated anti-rat IgG (BA-4001, Vector Laboratories) diluted 1:100 in PBS containing 1% BSA for 45 min at room temperature. Following treatment with Avidin/Biotin blocking solution (Avidin/Biotin Blocking Kit, Sp-2001, Vector Laboratories) to inhibit endogenous peroxidase activity, the secondary antibody was linked via streptavidin to a biotinylated peroxidase (POD) according to the manufacturer’s instructions (Vectorstain ABC Kit, Peroxidase Standard PK-4000, Vector Laboratories). Antigens were visualized via POD using the chromogen 3,3′- Diaminobenzidin (DAB) (Kem-En-Tec Diagnostics). For quantification of immune cells identical brain sections (thickness 10 µm) at the level of the basal ganglia (0.5 mm anterior from bregma) were selected and cell counting was performed from 5 subsequent slices (distance 100 µm) from 4 different animals.

### Apoptosis measurement

Apoptotic neurons in the ischemic hemisphere 24 h after tMCAO were visualized by TUNEL on cryo-embedded slices. Brain slices (10 µm thickness) were fixed in acetone for 10 min and blocked for 1 h in 5% BSA in PBS containing 1% Goat Serum and 0.3% Triton to prevent nonspecific binding. A mouse antibody to NeuN (MAB377, Millipore, 1:1000 in PBS) was applied over night at 4 °C. Proteins were detected by 45 min of incubation with Dylight 488–conjugated goat antibody to mouse secondary antibodies (polyclonal, ab96871, Abcam) at a dilution of 1:200 in 1% BSA in PBS. TUNEL positive cells were stained using the in situ cell death detection kit TMR red (Roche 12 156 792 910) following the manual instructions. Negative controls included omission of primary or secondary antibody and gave no signals (not shown).

### Western blot analysis

Brains were extracted from sacrificed stroke animals and classified as ipsilateral and contralateral. These were fast-frozen in liquid nitrogen, then stored at −80 °C until the time of analysis. Snap-frozen brains were crunched in liquid nitrogen and the brain powder was transferred into an eppendorf tube. Then hot Laemmli buffer (Bio-Rad, Veenendaal, The Netherlands), pre-heated at 95 °C and containing 5% β-mercaptoethanol, was added and the powder was lysed and denaturated for 10 min at 65 °C. Ultrasounds (Hielscher, Teltow, Germany) were used to homogenize the samples and then they were centrifuged at 12,000×*g* for 15 min at 4 °C. The supernatant was collected and stored at −80 °C. The total protein concentration was measured in the tissue supernatant by using the RC DC assay (Bio-Rad, Veenendaal, The Netherlands). Brain lysates were separated on a NuPAGE Novex 10% Bis–Tris Midi Gel 1.0 mm × 26 well, (Novex Life Technologies, Bleiswijk, The Netherlands) with the XCell SureLock Midi-Cell running tank (Life Technologies, Bleiswijk, The Netherlands). According to the invitrogen protocol MES running buffer was used (Life Technologies, Bleiswijk, The Netherlands).15 µg protein per well were loaded and laemmli-buffer containing plus 5% β-mercaptoethanol was used to adjust the volume and 0.3 µl protein marker were applied (LI-COR IRDye protein molecular weight marker, Licor Westburg, Leusden, The Netherlands). Proteins were transferred to nitrocellulose membranes using NuPAGE Iblot Gel Transfer Stacks Nitrocellulose (Novex Life Technologies, Bleiswijk, The Netherlands) on the iBlot Gel Transfer Device (Life Technologies, Bleiswijk, The Netherlands). The blot was stained with Ponceau S (Sigma Aldrich, Zwijndrecht, The Netherlands) to check for complete protein transfer. Non-specific reactivity was blocked by incubating the membranes for 1 h at room temperature with blocking buffer, 1 part Odyssey buffer (Licor Westburg, Leusden, The Netherlands) and 1 part PBS (Merck Chemicals, Amsterdam, The Netherlands). Hybridization also took place in Odyssey-PBS Blocking Buffer. The membranes were incubated with primary antibody overnight at 4 °C with a self-made polyclonal rabbit sGC-α1 antibody (dilution 1:2000) or with a polyclonal rabbit sGC-β1 antibody (dilution 1:2000). After three washing steps with PBS containing 0.1% Tween-20 (Merck Chemicals, Amsterdam, The Netherlands), the membranes were incubated with the secondary antibody for 1 h at room temperature with the secondary antibody (1:25,000 dilution, donkey anti rabbit CW800). Then the membranes were washed again 3 times with PBS-0.1%T and three times with PBS. Reactive proteins were visualized using a near-infrared imager (Oddyssey detection system, Licor Westburg, Leusden, The Netherlands) and the protein levels were determined by densitometric analysis of the specific protein bands (Image J). GAPDH (Millipore, Merck Chemicals, Amsterdam, The Netherlands) was used as an internal control. Actin or GAPDH served as loading control for all Western blot experiments. After normalization to loading control, sGCα1 and b1 protein data were expressed as mean fold change relative to the concentration in healthy brain tissue, which was set to 1.

### PCR studies

Total RNA was prepared with a Miccra D-8 power homogenizer (ART) using the TRIzol reagent® (Invitrogen) and was quantified spectrophotometrically. Then, 1 µg of total RNA were reversely transcribed with the TaqMan® Reverse Transcription Reagents (Applied Biosystems) according to the manufacturer’s protocol using random hexamers. Relative gene expression levels of interleukin(Il)-1ß (assay ID: Mm 00434228_m1, Applied Biosystems) and tumor necrosis factor(Tnf)α (assay ID: Mm 00443258_m1, Applied Biosystems) were quantified with the fluorescent TaqMan® technology. Gapdh (TaqMan® Predeveloped Assay Reagents for gene expression, part number: 4352339E, Applied Biosystems) was used as an endogenous control to normalize the amount of sample RNA. The PCR was performed with equal amounts of cDNA in the StepOnePlusTM Real-Time PCR System (Applied Biosystems) using the TaqMan® Universal 2× PCR Master Mix (Applied Biosystems). Reactions (total volume 12.5 µl) were incubated at 50 °C for 2 min, at 95 °C for 10 min followed by 40 cycles of 15 s at 95 °C and 1 min at 60 °C. Water controls were included to ensure specificity. Each sample was measured in triplicate and data points were examined for integrity by analysis of the amplification plot. The comparative Ct method was used for relative quantification of gene expression.

### Rat hippocampal slice experiments

MTT (3-(4,5-dimethylthiazol-2-yl)-diphenyltetrazolim bromide) from Sigma (Madrid, Spain), KT5823 from Tocris (Biogen Científica, Madrid, Spain) and BAY60-2770 as well as BAY58-2776 from Maastricht University (The Netherlands). Adult male Sprague-Dawley rats (275–325 g) from a colony of our animal quarters were used. Experimental procedures were approved by the institutional Ethics Committee of Universidad Autónoma de Madrid (Spain) in accordance with the European Guidelines for the use and care of animals for research. All efforts were made in order to reduce animal suffering and decrease the number of animals used. For hippocampal slices preparation and induction of oxygen and glucose deprivation (OGD), animals were decapitated under sodium pentobarbital anesthesia (60 mg/kg, i.p.), the brains were removed and located into ice-cold Krebs bicarbonate dissection buffer (pH 7.4) composed of: NaCl 120 mM, KCl 2 mM, NaHCO_3_ 26 mM, KH_2_PO_4_ 1.18 mM, MgSO_4_ 10 mM, CaCl 0.5 mM, glucose 11 mM and sucrose 200 mM. The solutions were pre-bubbled with 95% O_2_/5% CO_2_ for not less than 30 min before starting the experiment. Thereafter, hippocampi were dissected and immersed in cold, oxygenated dissection buffer before sectioning in transverse slices of 250 µM using a McIlwain Tissue Chopper. Attempting to stabilize tissue after slicing trauma, slices were transferred to sucrose-free dissection buffer, bubbled with 95% O_2_/5% CO_2_, during 45 min at 34 °C. Then, control group slices were incubated 15 min in a Krebs solution, containing (in mM): NaCl 120, KCl 2, NaHCO_3_ 26, CaCl 2, KH_2_PO_4_ 1.18, MgSO_4_ 1.19 and glucose 11 mM, which was equilibrated with 95% O_2_/5% CO_2._ Slices subjected to oxygen and glucose deprivation were incubated in a Krebs buffer pre-bubbled with 95% N_2_/5% CO_2_, where 2-deoxyglucose replaced glucose. OGD period was followed by 120 min of reoxygenation at 37 °C where slices were returned back to an oxygenated Krebs solution containing glucose. During the reoxygenation period, slices were treated with different concentrations of BAY60-2770 or BAY58-2667. KT-5823 was used as an inhibitor of PKG. Cellular viability was quantified by their ability to reduce MTT.^60^ After the reoxygenation period, slices were incubated with MTT (0.5 mg/ml) in Krebs bicarbonate solution for 40 min at 37 °C. Cell active dehydrogenases can cleave the tetrazolium ring of MTT in order to generate a precipitated formazan. The formazan produced was solubilized by adding 200 µl dimethyl sulfoxide (DMSO), giving rise to a colored compound whose optical density was measured in an ELISA microplate reader at 540 nm. Absorbance values obtained in control slices were taken as 100% viability.

### Statistical analysis

All results obtained from stroke brains were analyzed using the GraphPad Prism 5.0 software (GraphPad Software Inc., San Diego, CA, USA). Data were expressed as the means ± standard error of the mean of separate experiments (*n* = 4). Statistical comparisons between groups were performed using one-way ANOVA, followed by a two-tailed unpaired Student's *t* test (for sGC alpha1 subunit). Differences between two treatments were considered significant at *P* < 0.05. All results were expressed as mean ± SEM except for ordinal functional outcome scales that were depicted as scatter plots. Numbers of animals (N = 10) necessary to detect a standardized effect size on infarct volumes ≥ 0.2 (vehicle treated control mice vs. BAY60-2770 treated mice) were determined via a priori sample size calculation with the following assumptions: α = 0.05, ß = 0.2, mean, 20% SD of the mean (GraphPad Stat Mate 2.0; GraphPad Software). Data were tested for Gaussian distribution with the D’Agostino and Pearson omnibus normality test and then analyzed by one-way analysis of variance (ANOVA) with posthoc Bonferroni adjustment for *P* values. If only 2 groups were compared, unpaired, two-tailed Student's *t* test was applied. Nonparametric functional outcome scores were compared by Kruskal–Wallis test with posthoc Dunn multiple comparison test. For comparison of survival curves the log-rank test was used. *P* values < 0.05 were considered statistically significant.

### Data availability

Experimental data from the cGMP-related cluster within the human diseasome are available in the supplemental tables S1,S2A–D and S3 from the authors. Relevant experimental data are available from the authors.

## Electronic supplementary material

Supplemental Material

## Electronic supplementary material

**Supplementary information** accompanies the paper on the *npj Systems Biology and Applications* website (10.1038/s41540-017-0039-7).
