# Supplementary material for: A diseasome cluster-based drug repurposing of soluble guanylate cyclase activators from smooth muscle relaxation to direct neuroprotection
Source: NPJ Syst Biol Appl. 2018 Feb 5;4:8. doi: 10.1038/s41540-017-0039-7 (PMC5799370; doi:10.1038/s41540-017-0039-7)
Supplement: Supplementary file 1 — Supplemental Material [file 41540_2017_39_MOESM1_ESM.docx]

*NPJ | Systems Biology and Applications* – *Article*

A diseasome cluster-based drug repurposing of soluble guanylate cyclase activators from smooth muscle relaxation to direct neuroprotection

Friederike Langhauser^1,*^, Ana I. Casas^2,3,*^, Vu-Thao-Vi Dao^2^, Emre Guney^4,5^, Jörg Menche^6^, Eva Geuss^1^, Pamela W.M. Kleikers^2^, Manuela G. López^3^, Albert-L. Barabási^4,5,7,8^, Christoph Kleinschnitz^1,9,**^ & Harald H.H.W. Schmidt^2,**^

^1^Department of Neurology, University Hospital Würzburg, Josef-Schneider-Straße 11, 97080 Würzburg, Germany; ^2^Department of Pharmacology & Personalised Medicine, CARIM, Maastricht University, Universiteitssingel 50, 6229 ER Maastricht, The Netherlands; ^3^Departamento de Farmacología, Facultad de Medicina, Universidad Autónoma de Madrid, Arzobispo Morcillo s/n, 28029 Madrid, Spain; ^4^Center for Complex Network Research (CCNR) and Department of Physics, Northeastern University, Boston, MA 02115, USA; ^5^Center for Cancer Systems Biology (CCSB) and Department of Cancer Biology, Dana-Farber Cancer Institute, Harvard Medical School, Boston, MA 02215, USA; ^6^CeMM Research Center for Molecular Medicine of the Austrian Academy of Sciences, Lazarettgasse 14 AKH BT25.3, 1090 Vienna, Austria; ^7^Department of Medicine, Brigham and Women’s Hospital, Harvard Medical School, Boston, MA 02115, USA; ^8^Center for Network Science, Central European University, Nador u. 9, 1051 Budapest, Hungary; ^9^Department of Neurology, University Hospital Essen, Hufelandstraße 55 D-45147 Essen, Germany. ^*^These authors contributed equally to this work. ^**^These senior authors contributed equally to this work.

^*^Correspondence: Harald H.H. W. Schmidt ([h.schmidt@maastrichtuniversity.nl)](mailto:h.schmidt@maastrichtuniversity.nl)) or to Ana I. Casas ([a.casasguijarro@maastrichtuniversity.nl)](mailto:a.casasguijarro@maastrichtuniversity.nl))

Running title: Repurposing of soluble guanylate cyclase activators to direct neuroprotection.

**SUPPLEMENTARY FIGURES**

**
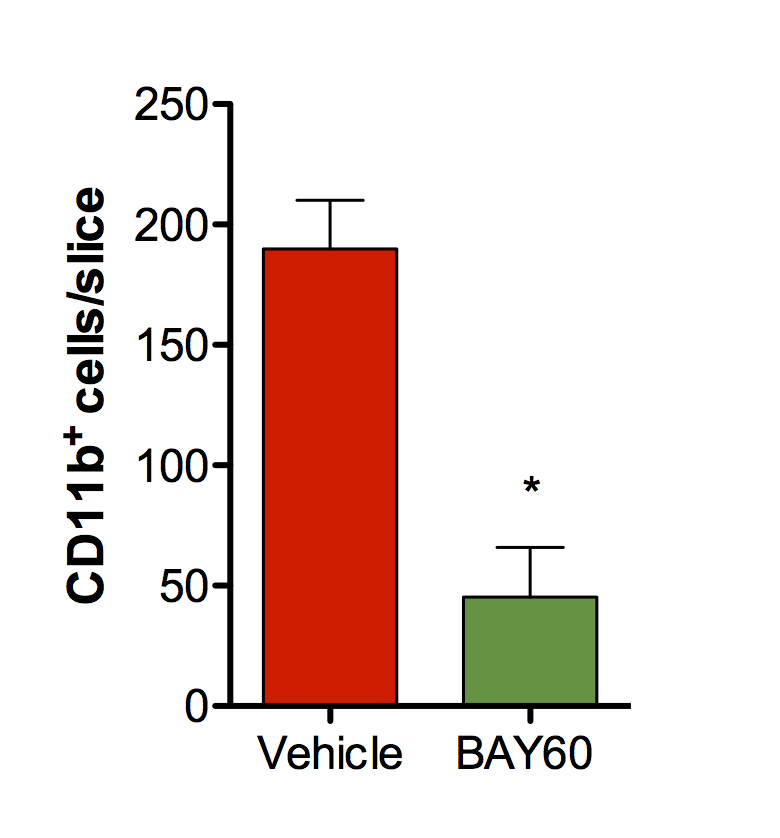
**

**Fig. S1. Post-stroke treatment with BAY60-2770 reduces microglia activation and infiltration of macrophages.** 24h after transient occlusion of the middle cerebral artery (tMCAO) microglia activation/infiltration of macrophages is reduced in mice treated with BAY60-2770 (10$\mu$g/kg) 1h post-stroke (n=4, p<0,05).

**Fig. S2. Post-stroke treatment with BAY60-2770 down-regulates IL-1ß and TNF-**$\boldsymbol{\alpha}$ **gene expression.** 24h post transient occlusion of the middle cerebral artery (tMCAO) (**A**) IL-1ß is reduced in mice treated with BAY60-2770 (10$\mu$g/kg) both 1h (n=6, p<0,01) and 4h post-stroke (n=4, p<0,01). (**B**) TNF-$\alpha$ gene expression was reduced in animals treated 4h post-stroke while no effect was detected when treated after 1 h (n=4, p<0,05).

**SUPPLEMENTARY TABLES**

| **Table S1.** The coverage and average degree of diseasomes generated based on gene overlap, overlap of the proteins connecting disease genes, symptom similarity and comorbidity. | | | |
| --- | --- | --- | --- |
| Data source | # of nodes | # of edges | Average degree |
| Genes | 12 | 23 | 3.83 |
| Interactions | 12 | 42 | 7 |
| Symptoms | 8 | 7 | 1.75 |
| Comorbidity | 9 | 27 | 6 |

**Table S2A.** Scores based on the number of genes shared by pairs of diseases in the highlighted diseasome cluster.

| Pathophenotype 1 | Pathophenotype 2 | Score | Genes |
| --- | --- | --- | --- |
| Alzheimer Disease | Dementia | 36 | 339761\|5663\|10452\|274\|8301\|5664\|283226\|10347\|5071\|7305\|1191\|4535\|4536\|51435\|344558\|643680\|10913\|25902\|1378\|348\|341\|23607\|54209\|2629\|9846\|23036\|9627\|64231\|55103\|2041\|945\|5819\|51338\|351\|338399\|4973 |
| Myocardial Infarction | Stroke | 21 | 100130239\|10151\|7804\|375056\|6597\|7809\|170690\|283652\|255738\|6387\|114036\|241\|63951\|55759\|1952\|79192\|100048912\|221692\|9992\|23022\|4973 |
| Dementia | Stroke | 13 | 339761\|23209\|9445\|25902\|4535\|5663\|341\|351\|5664\|5654\|4854\|348\|4973 |
| Hypertension | Stroke | 12 | 7369\|7917\|51196\|728553\|143872\|128609\|183\|79614\|3077\|128611\|490\|51320 |
| Alzheimer Disease | Stroke | 9 | 339761\|4535\|25902\|5663\|341\|351\|5664\|348\|4973 |
| Atherosclerosis | Stroke | 9 | 54984\|168455\|100130537\|5294\|341\|1909\|22882\|348\|347704 |
| Diabetes Mellitus, Type 2 | Stroke | 9 | 3667\|57118\|3784\|677828\|4535\|100130239\|8622\|63951\|4853 |
| Dementia | Parkinson Disease | 5 | 9627\|2629\|5071\|6622\|4137 |
| Alzheimer Disease | Parkinson Disease | 3 | 2629\|5071\|9627 |
| Asthma | Stroke | 2 | 4088\|5144 |
| Alzheimer Disease | Atherosclerosis | 2 | 348\|341 |
| Heart Failure | Stroke | 2 | 9960\|771 |
| Atherosclerosis | Dementia | 2 | 348\|341 |
| Diabetes Mellitus, Type 2 | Myocardial Infarction | 2 | 100130239\|63951 |
| Asthma | Parkinson Disease | 1 | 3122 |
| Asthma | Diabetes Mellitus, Type 2 | 1 | 169026 |
| Alzheimer Disease | Myocardial Infarction | 1 | 4973 |
| Alzheimer Disease | Diabetes Mellitus, Type 2 | 1 | 4535 |
| Obesity | Stroke | 1 | 4094 |
| Migraine Disorders | Stroke | 1 | 4035 |
| Dementia | Myocardial Infarction | 1 | 4973 |
| Diabetes Mellitus, Type 2 | Obesity | 1 | 79068 |
| Dementia | Diabetes Mellitus, Type 2 | 1 | 4535 |
| Alzheimer Disease | Asthma | 0 |  |
| Asthma | Heart Failure | 0 |  |
| Asthma | Atherosclerosis | 0 |  |
| Asthma | Hypertension | 0 |  |
| Asthma | Myocardial Infarction | 0 |  |
| Asthma | Obesity | 0 |  |
| Asthma | Dementia | 0 |  |
| Asthma | Migraine Disorders | 0 |  |
| Alzheimer Disease | Heart Failure | 0 |  |
| Alzheimer Disease | Hypertension | 0 |  |
| Alzheimer Disease | Obesity | 0 |  |
| Alzheimer Disease | Migraine Disorders | 0 |  |
| Atherosclerosis | Heart Failure | 0 |  |
| Heart Failure | Hypertension | 0 |  |
| Heart Failure | Parkinson Disease | 0 |  |
| Heart Failure | Myocardial Infarction | 0 |  |
| Heart Failure | Obesity | 0 |  |
| Dementia | Heart Failure | 0 |  |
| Heart Failure | Migraine Disorders | 0 |  |
| Diabetes Mellitus, Type 2 | Heart Failure | 0 |  |
| Atherosclerosis | Hypertension | 0 |  |
| Atherosclerosis | Parkinson Disease | 0 |  |
| Atherosclerosis | Myocardial Infarction | 0 |  |
| Atherosclerosis | Obesity | 0 |  |
| Atherosclerosis | Migraine Disorders | 0 |  |
| Atherosclerosis | Diabetes Mellitus, Type 2 | 0 |  |
| Parkinson Disease | Stroke | 0 |  |
| Hypertension | Parkinson Disease | 0 |  |
| Hypertension | Myocardial Infarction | 0 |  |
| Hypertension | Obesity | 0 |  |
| Dementia | Hypertension | 0 |  |
| Hypertension | Migraine Disorders | 0 |  |
| Diabetes Mellitus, Type 2 | Hypertension | 0 |  |
| Myocardial Infarction | Parkinson Disease | 0 |  |
| Obesity | Parkinson Disease | 0 |  |
| Migraine Disorders | Parkinson Disease | 0 |  |
| Diabetes Mellitus, Type 2 | Parkinson Disease | 0 |  |
| Myocardial Infarction | Obesity | 0 |  |
| Migraine Disorders | Myocardial Infarction | 0 |  |
| Dementia | Obesity | 0 |  |
| Migraine Disorders | Obesity | 0 |  |
| Dementia | Migraine Disorders | 0 |  |
| Diabetes Mellitus, Type 2 | Migraine Disorders | 0 |  |

ENTREZ GeneIds are provided for the genes in common

**Table S2B.** Scores (z-score) based on the number of interactions between disease genes for each pair of disease in the highlighted diseasome cluster.

| Pathophenotype 1 | Pathophenotype 2 | Score | Interacting genes |
| --- | --- | --- | --- |
| Alzheimer Disease | Dementia | 35,66358 | 5663-5664\|5663-5663\|5663-348\|5663-4137\|5663-351\|5663-4854\|10452-10452\|10452-351\|274-274\|8301-8301\|5664-5664\|5664-5663\|5664-351\|5664-4854\|5071-5071\|5071-6622\|5071-9627\|5071-4137\|7305-7305\|7305-54209\|1191-1191\|1191-5621\|1191-351\|4535-4535\|4535-4536\|4536-4535\|4536-4536\|51435-51435\|344558-344558\|10913-10913\|25902-25902\|23036-23036\|348-5663\|348-5621\|348-348\|348-4137\|348-351\|341-341\|23607-23607\|54209-7305\|54209-54209\|2629-2629\|9846-9846\|1378-1378\|9627-5071\|9627-6622\|9627-9627\|55103-55103\|2041-2041\|945-945\|5819-5819\|351-5664\|351-5663\|351-10452\|351-6622\|351-1191\|351-5621\|351-348\|351-9445\|351-4137\|351-351\|4973-4973 |
| Dementia | Parkinson Disease | 14,57167 | 7345-351\|5071-5071\|5071-6622\|5071-9627\|5071-4137\|6622-5071\|6622-6622\|6622-9627\|6622-4137\|6622-351\|120892-6622\|4724-4535\|4724-4536\|4905-351\|7054-6622\|2629-2629\|27429-351\|9627-5071\|9627-6622\|9627-9627\|4137-5663\|4137-5071\|4137-6622\|4137-348\|4137-4137\|4137-351\|65018-5071 |
| Alzheimer Disease | Parkinson Disease | 11,49063 | 5663-4137\|5071-5071\|5071-6622\|5071-9627\|5071-4137\|5071-65018\|4535-4724\|4536-4724\|348-4137\|2629-2629\|9627-5071\|9627-6622\|9627-9627\|351-7345\|351-6622\|351-4905\|351-27429\|351-4137 |
| Dementia | Stroke | 8,56246 | 23209-23209\|3717-9846\|341-341\|348-5663\|348-5621\|348-348\|348-4137\|348-351\|7804-1191\|7804-348\|9709-5663\|7046-1191\|4035-348\|4035-351\|1495-5663\|7048-1191\|29994-5621\|8870-5621\|1829-5663\|2316-5664\|2316-5663\|6654-274\|6654-23607\|1804-5621\|4973-4973\|3827-351\|775-274\|284217-351\|5664-5664\|5664-5663\|5664-351\|5664-4854\|2099-348\|4978-5621\|2934-351\|4541-4535\|4541-4536\|2161-351\|4340-5621\|84898-5663\|5663-5664\|5663-5663\|5663-348\|5663-4137\|5663-351\|5663-4854\|4854-5664\|4854-5663\|4854-4854\|4638-23209\|7917-5621\|84441-4854\|4535-4535\|4535-4536\|182-4854\|652-5654\|5781-945\|5781-9846\|7414-5071\|2581-2629\|25902-25902\|5654-5654\|4088-1378\|5340-5621\|4524-25902\|3728-5663\|4089-4535\|5444-1191\|7428-25978\|3106-10452\|3106-23435\|65125-1191\|5921-3064\|4205-4536\|1278-351\|351-5664\|351-5663\|351-10452\|351-6622\|351-1191\|351-5621\|351-348\|351-9445\|351-4137\|351-351\|9445-9445\|9445-351\|1277-5654\|1284-351\|1281-5654\|1282-351\|4853-5664\|4853-5663\|4851-5664\|4851-5663\|4851-4854 |
| Atherosclerosis | Dementia | 7,899962 | 341-341\|348-5663\|348-5621\|348-348\|348-4137\|348-351 |
| Alzheimer Disease | Stroke | 7,182523 | 5663-348\|5663-9709\|5663-1495\|5663-1829\|5663-2316\|5663-5664\|5663-84898\|5663-5663\|5663-4854\|5663-3728\|5663-351\|5663-4853\|5663-4851\|10452-3106\|10452-351\|274-6654\|274-775\|5664-2316\|5664-5664\|5664-5663\|5664-4854\|5664-351\|5664-4853\|5664-4851\|5071-7414\|1191-7804\|1191-7046\|1191-7048\|1191-5444\|1191-65125\|1191-351\|4535-4541\|4535-4535\|4535-4089\|4536-4541\|4536-4535\|4536-4205\|25902-25902\|25902-4524\|348-348\|348-7804\|348-4035\|348-2099\|348-5663\|348-351\|341-341\|23607-6654\|2629-2581\|9846-3717\|9846-5781\|1378-4088\|945-5781\|351-348\|351-4035\|351-3827\|351-284217\|351-5664\|351-2934\|351-2161\|351-5663\|351-1278\|351-351\|351-9445\|351-1284\|351-1282\|4973-4973 |
| Alzheimer Disease | Atherosclerosis | 6,971922 | 5663-348\|348-348\|341-341\|351-348 |
| Heart Failure | Migraine Disorders | 5,594309 | 9960-4035 |
| Atherosclerosis | Stroke | 4,779799 | 54984-7015\|54984-54984\|54984-4205\|5294-4893\|5294-3845\|5294-2934\|5294-5781\|5294-5294\|5294-4851\|341-341\|1909-1909\|22882-22882\|348-348\|348-7804\|348-4035\|348-2099\|348-5663\|348-351 |
| Atherosclerosis | Migraine Disorders | 3,439019 | 348-4035 |
| Myocardial Infarction | Stroke | 3,136032 | 255738-255738\|348-7804\|7804-7804\|7809-7809\|1952-1952\|55759-55759\|4973-4973\|6597-6597\|6387-6387\|9992-9992\|2099-6597\|9722-7804\|241-241\|3784-9992\|4088-6597\|88-23022\|4089-6597\|375056-375056\|221692-221692\|3757-9992\|23022-23022\|4851-55759 |
| Alzheimer Disease | Diabetes Mellitus, Type 2 | 2,926575 | 5663-6934\|5663-4853\|5664-4853\|5664-54536\|4535-4535\|4536-4535\|25902-9861\|9846-640\|351-9479\|351-54536 |
| Heart Failure | Parkinson Disease | 2,923854 | 9960-1981 |
| Diabetes Mellitus, Type 2 | Stroke | 2,895556 | 3759-3767\|10060-3767\|8622-8622\|3717-7297\|3717-3667\|3717-3643\|5629-3172\|3753-3784\|7804-9479\|4035-9479\|2316-4544\|4893-2888\|4878-3172\|57118-57118\|595-4760\|5664-4853\|5664-54536\|9992-3784\|6263-6927\|2099-3172\|51196-3636\|2261-116985\|23007-3636\|2200-10253\|3667-7297\|3667-3667\|3667-3643\|3667-2888\|3784-3784\|3784-9861\|4541-4535\|5583-3667\|5583-3643\|780-3667\|5573-6517\|2161-3172\|10142-3784\|5663-6934\|5663-4853\|84441-4853\|4535-4535\|859-3643\|182-4853\|5781-10253\|5781-3667\|5781-3643\|25902-9861\|5894-10253\|5894-3643\|4088-3172\|221421-10363\|80271-3636\|80271-4853\|3728-6934\|4089-10363\|4089-4535\|4089-3172\|2212-640\|3184-10644\|5350-5506\|3106-9861\|3107-9861\|65125-7466\|65125-8567\|5921-9223\|5921-3643\|771-9905\|351-9479\|351-54536\|2160-3172\|4853-4853 |
| Hypertension | Stroke | 2,87462 | 490-490\|8870-7917\|4893-51196\|3827-183\|7369-7369\|51196-51196\|23007-51196\|7917-7917\|183-183\|3077-3077\|80271-51196\|472-7917 |
| Alzheimer Disease | Myocardial Infarction | 2,867633 | 1191-7804\|348-7804\|4973-4973 |
| Dementia | Obesity | 2,776977 | 4685-5621\|3952-1191\|5468-1191\|5468-5621 |
| Dementia | Diabetes Mellitus, Type 2 | 2,518384 | 5664-4853\|5664-54536\|5663-6934\|5663-4853\|3064-4760\|6622-9479\|4535-4535\|4536-4535\|25902-9861\|9846-640\|351-9479\|351-54536 |
| Asthma | Dementia | 2,423017 | 4088-1378\|1017-5663\|1017-25902\|1017-4137\|85480-5621\|4855-5664\|4855-5663 |
| Atherosclerosis | Myocardial Infarction | 2,395912 | 348-7804 |
| Alzheimer Disease | Migraine Disorders | 2,375425 | 348-4035\|351-4035 |
| Asthma | Stroke | 2,313601 | 4088-6736\|4088-7046\|4088-7048\|4088-2316\|4088-6597\|4088-2099\|4088-1874\|4088-4088\|4088-4089\|4088-5054\|4088-4000\|4088-4851\|1017-595\|1017-2099\|1017-5583\|1017-5573\|1017-4626\|1017-5663\|1017-25902\|1017-4088\|1017-51530\|1017-3184\|1017-7428\|1017-4000\|1017-5932\|5144-5144\|5089-1290\|3560-3717\|3560-5894\|1999-7048\|1999-337880\|1999-10142\|4855-5664\|4855-5663\|4855-84441\|4855-4088\|4855-4089 |
| Alzheimer Disease | Asthma | 2,128399 | 4088-1378\|1017-5663\|1017-25902\|4855-5663\|4855-5664 |
| Dementia | Myocardial Infarction | 1,956644 | 7804-1191\|7804-348\|4973-4973 |
| Dementia | Migraine Disorders | 1,80817 | 348-4035\|351-4035 |
| Alzheimer Disease | Obesity | 1,680616 | 1191-3952\|1191-5468 |
| Migraine Disorders | Stroke | 1,664181 | 348-4035\|7046-338567\|4035-4035\|7048-338567\|6640-6323\|9722-4035\|9960-4035\|4088-63976\|5054-4035\|351-4035 |
| Myocardial Infarction | Obesity | 1,296534 | 6597-5468 |
| Diabetes Mellitus, Type 2 | Heart Failure | 1,238739 | 771-9905 |
| Heart Failure | Stroke | 1,190938 | 9960-4035\|9960-9960\|771-771 |
| Asthma | Diabetes Mellitus, Type 2 | 1,153179 | 4088-3172\|3570-9223\|1017-9055\|169026-169026\|3119-3630\|3118-3630\|1999-9861 |
| Atherosclerosis | Parkinson Disease | 1,133333 | 348-4137 |
| Diabetes Mellitus, Type 2 | Parkinson Disease | 0,962424 | 55737-4544\|55737-9559\|6622-9479\|4724-4535\|6239-10363\|6239-4760 |
| Dementia | Hypertension | 0,817393 | 7917-5621 |
| Asthma | Parkinson Disease | 0,784118 | 3122-3122\|1017-1981\|1017-4137 |
| Asthma | Migraine Disorders | 0,645382 | 4088-63976 |
| Diabetes Mellitus, Type 2 | Myocardial Infarction | 0,35079 | 7804-9479\|9992-3784 |
| Diabetes Mellitus, Type 2 | Obesity | 0,32138 | 8431-3172\|79068-79068 |
| Diabetes Mellitus, Type 2 | Hypertension | 0,319186 | 51196-3636 |
| Parkinson Disease | Stroke | 0,246077 | 348-4137\|7046-57111\|4541-4724\|9960-1981\|5663-4137\|4535-4724\|7414-5071\|2581-2629\|4089-57111\|3184-1981\|7428-55737\|7428-7345\|3106-55737\|351-7345\|351-6622\|351-4905\|351-27429\|351-4137 |
| Asthma | Myocardial Infarction | 0,203394 | 4088-6597 |
| Diabetes Mellitus, Type 2 | Migraine Disorders | 0,084172 | 4035-9479 |
| Heart Failure | Hypertension | -0,127515 |  |
| Atherosclerosis | Heart Failure | -0,135388 |  |
| Heart Failure | Obesity | -0,172818 |  |
| Atherosclerosis | Hypertension | -0,219786 |  |
| Heart Failure | Myocardial Infarction | -0,223585 |  |
| Hypertension | Migraine Disorders | -0,247681 |  |
| Alzheimer Disease | Heart Failure | -0,288675 |  |
| Asthma | Heart Failure | -0,311017 |  |
| Hypertension | Obesity | -0,326063 |  |
| Atherosclerosis | Obesity | -0,334107 |  |
| Hypertension | Myocardial Infarction | -0,365846 |  |
| Dementia | Heart Failure | -0,378404 |  |
| Migraine Disorders | Myocardial Infarction | -0,396858 |  |
| Migraine Disorders | Obesity | -0,400734 |  |
| Obesity | Stroke | -0,415525 | 6597-5468\|6662-4094\|2099-8431\|1879-5079\|4094-4094 |
| Alzheimer Disease | Hypertension | -0,493086 |  |
| Hypertension | Parkinson Disease | -0,504291 |  |
| Asthma | Atherosclerosis | -0,528074 |  |
| Asthma | Hypertension | -0,570673 |  |
| Migraine Disorders | Parkinson Disease | -0,585491 |  |
| Atherosclerosis | Diabetes Mellitus, Type 2 | -0,675805 |  |
| Obesity | Parkinson Disease | -0,686623 |  |
| Myocardial Infarction | Parkinson Disease | -0,72786 |  |
| Asthma | Obesity | -0,797798 |  |

Interacting genes are given by their ENTREZ GeneIds

**Table S2C.** Scores (Jaccard Index) based on the number of symptoms shared by pairs of diseases in the highlighted diseasome cluster.

| Pathophenotype 1 | Pathophenotype 2 | Score | Symptoms | | | |
| --- | --- | --- | --- | --- | --- | --- |
| Alzheimer Disease | Dementia | 0,731034 | aphasia, broca\|mobility limitation\|hearing disorders\|diarrhea\|gait disorders, neurologic\|weight loss\|echolalia\|hypothermia\|urinary bladder, neurogenic\|syncope, vasovagal\|aphasia, primary progressive\|illusions\|syncope\|mutism\|thinness\|weight gain\|memory disorders\|persistent vegetative state\|prosopagnosia\|aphasia\|hallucinations\|auditory perceptual disorders\|nausea\|language development disorders\|urinary bladder, overactive\|obesity\|language disorders\|lethargy\|anomia\|supranuclear palsy, progressive\|hemianopsia\|cachexia\|catatonia\|pain\|snoring\|psychomotor agitation\|hypercapnia\|psychomotor disorders\|muscular atrophy\|flushing\|confusion\|dyslexia, acquired\|vomiting\|apraxia, ideomotor\|hypokinesia\|vision disorders\|coma\|tremor\|dizziness\|dysarthria\|paralysis\|hyperkinesis\|hearing loss\|muscle weakness\|mental retardation\|delirium\|anoxia\|gait apraxia\|sleep disorders\|sleep deprivation\|communication disorders\|taste disorders\|body weight\|ataxia\|apraxias\|anorexia\|seizures\|dyslexia\|unconsciousness\|amnesia\|myoclonus\|psychophysiologic disorders\|headache\|learning disorders\|speech disorders\|agnosia\|aging, premature\|blindness\|hyperphagia\|cerebellar ataxia\|muscle rigidity\|amnesia, transient global\|hemiplegia\|amnesia, retrograde\|akathisia, drug-induced\|reflex, abnormal\|articulation disorders\|olfaction disorders\|primary progressive nonfluent aphasia\|paraparesis, spastic\|agraphia\|urinary incontinence, urge\|ophthalmoplegia, chronic progressive external\|chorea\|neurobehavioral manifestations\|fever\|low back pain\|korsakoff syndrome\|fatigue\|gerstmann syndrome\|color vision defects\|nocturia\|dystonia\|perceptual disorders\|aphasia, wernicke\|urinary incontinence | | | |
| Dementia | Parkinson Disease | 0,565934 | aphasia, broca\|mobility limitation\|urinary incontinence, urge\|weight loss\|echolalia\|urinary bladder, neurogenic\|hypothermia\|muscle spasticity\|illusions\|syncope\|thinness\|athetosis\|memory disorders\|paraplegia\|stuttering\|aphasia\|hallucinations\|auditory perceptual disorders\|nausea\|quadriplegia\|urinary bladder, overactive\|weight gain\|language disorders\|anomia\|supranuclear palsy, progressive\|eye manifestations\|catatonia\|snoring\|psychomotor agitation\|hypercapnia\|muscle hypertonia\|vertigo\|psychomotor disorders\|muscular atrophy\|flushing\|anorexia\|confusion\|asthenia\|vomiting\|apraxia, ideomotor\|hypokinesia\|vision disorders\|coma\|tremor\|obesity\|dyskinesias\|dysarthria\|paralysis\|hyperkinesis\|agraphia\|muscle weakness\|mental retardation\|delirium\|anoxia\|catalepsy\|gait apraxia\|sleep disorders\|sleep deprivation\|taste disorders\|body weight\|ataxia\|apraxias\|constipation\|seizures\|gait ataxia\|amnesia\|myoclonus\|psychophysiologic disorders\|headache\|learning disorders\|speech disorders\|agnosia\|gait disorders, neurologic\|hyperphagia\|cerebellar ataxia\|hearing loss, central\|sensation disorders\|muscle rigidity\|hemiplegia\|ophthalmoplegia\|akathisia, drug-induced\|reflex, abnormal\|articulation disorders\|olfaction disorders\|primary progressive nonfluent aphasia\|paresis\|diarrhea\|ophthalmoplegia, chronic progressive external\|chorea\|pain, intractable\|mental fatigue\|fever\|pain\|torticollis\|fatigue\|communication disorders\|color vision defects\|respiratory aspiration\|nocturia\|dizziness\|dystonia\|perceptual disorders\|urinary incontinence | | | |
| Hypertension | Myocardial Infarction | 0,551913 | edema, cardiac\|edema\|pain, postoperative\|weight loss\|hypothermia\|hyperoxia\|syncope\|thinness\|weight gain\|tremor\|memory disorders\|apnea\|paraplegia\|chest pain\|aphasia\|hypesthesia\|hallucinations\|nausea\|quadriplegia\|hemoptysis\|urinary bladder, overactive\|obesity\|neck pain\|albuminuria\|constipation\|cardiac output, low\|abdominal pain\|heartburn\|eye hemorrhage\|snoring\|psychomotor agitation\|hypercapnia\|spasm\|vertigo\|psychomotor disorders\|paresthesia\|flushing\|syncope, vasovagal\|birth weight\|confusion\|heart murmurs\|asthenia\|vomiting\|coma\|obesity, morbid\|cough\|dizziness\|facial paralysis\|paralysis\|hyperkinesis\|cyanosis\|muscle weakness\|delirium\|anoxia\|catalepsy\|sleep disorders\|sleep deprivation\|abdomen, acute\|proteinuria\|body weight\|dyspnea\|seizures\|unconsciousness\|pruritus\|hemianopsia\|purpura, schoenlein-henoch\|fetal hypoxia\|intermittent claudication\|eye manifestations\|psychophysiologic disorders\|headache\|ecchymosis\|dyspnea, paroxysmal\|blindness\|oliguria\|muscle rigidity\|amnesia, transient global\|hemiplegia\|angina, unstable\|reflex, abnormal\|angina pectoris\|purpura, thrombocytopenic\|hearing loss, sudden\|toothache\|paresis\|diarrhea\|hyperventilation\|angina pectoris, variant\|colic\|mental fatigue\|fever\|pain\|back pain\|overweight\|hypergammaglobulinemia\|fatigue\|hypoventilation\|acute coronary syndrome\|decerebrate state\|purpura, thrombotic thrombocytopenic\|facial pain | | | |
| Heart Failure | Myocardial Infarction | 0,544118 | hemianopsia\|sleep disorders\|edema, cardiac\|albuminuria\|sleep deprivation\|angina pectoris\|edema\|abdominal pain\|weight loss\|obesity\|psychophysiologic disorders\|hypothermia\|proteinuria\|body weight\|pain\|acute coronary syndrome\|spasm\|hypercapnia\|constipation\|seizures\|vertigo\|hyperoxia\|angina pectoris, variant\|syncope\|paresthesia\|cardiac output, low\|diarrhea\|hyperventilation\|fatigue\|thinness\|muscular atrophy\|fetal hypoxia\|syncope, vasovagal\|jaundice\|memory disorders\|headache\|birth weight\|purpura, thrombotic thrombocytopenic\|fever\|apnea\|respiratory sounds\|mental fatigue\|snoring\|confusion\|heart murmurs\|asthenia\|overweight\|dyspnea, paroxysmal\|vomiting\|intermittent claudication\|hiccup\|chest pain\|aphasia\|cachexia\|coma\|obesity, morbid\|oliguria\|cough\|hypoventilation\|dizziness\|nausea\|dyspepsia\|hemiplegia\|decerebrate state\|hemoptysis\|pruritus\|angina, unstable\|dyspnea\|muscle weakness\|abdomen, acute\|weight gain\|anoxia\|reflex, abnormal\|cyanosis | | | |
| Parkinson Disease | Stroke | 0,544041 | aphasia, broca\|mobility limitation\|edema\|weight loss\|reflex, babinski\|urinary bladder, neurogenic\|somatosensory disorders\|muscle spasticity\|illusions\|syncope\|muscle hypotonia\|shoulder pain\|phantom limb\|memory disorders\|tics\|paraplegia\|stuttering\|aphasia\|hallucinations\|auditory perceptual disorders\|nausea\|diplopia\|quadriplegia\|urinary bladder, overactive\|weight gain\|language disorders\|anomia\|snoring\|psychomotor agitation\|hypercapnia\|synkinesis\|spasm\|muscle hypertonia\|vertigo\|athetosis\|paresthesia\|muscular atrophy\|dyspnea\|confusion\|gagging\|vomiting\|apraxia, ideomotor\|hypokinesia\|vision disorders\|coma\|tremor\|cough\|obesity\|facial paralysis\|dyskinesias\|dysarthria\|paralysis\|vocal cord paralysis\|hyperkinesis\|agraphia\|muscle weakness\|delirium\|anoxia\|catalepsy\|sleep disorders\|hypothermia\|taste disorders\|body weight\|ataxia\|apraxias\|constipation\|seizures\|gait ataxia\|pupil disorders\|amnesia\|myoclonus\|headache\|speech disorders\|hyperalgesia\|agnosia\|gait disorders, neurologic\|cerebellar ataxia\|sensation disorders\|muscle rigidity\|hemiplegia\|ophthalmoplegia\|reflex, abnormal\|articulation disorders\|pseudobulbar palsy\|paresis\|urinary incontinence, urge\|chorea\|hyperventilation\|voice disorders\|pain, intractable\|mental fatigue\|cheyne-stokes respiration\|fever\|pain\|fatigue\|psychomotor disorders\|communication disorders\|hiccup\|respiratory aspiration\|nocturia\|dizziness\|dystonia\|perceptual disorders\|facial pain\|urinary incontinence | | | |
| Dementia | Stroke | 0,530303 | aphasia, broca\|mobility limitation\|weight loss\|urinary bladder, neurogenic\|aphasia, primary progressive\|muscle spasticity\|illusions\|syncope\|mutism\|athetosis\|memory disorders\|tinnitus\|persistent vegetative state\|paraplegia\|stuttering\|prosopagnosia\|aphasia\|hallucinations\|auditory perceptual disorders\|nausea\|language development disorders\|quadriplegia\|urinary bladder, overactive\|weight gain\|language disorders\|anomia\|hemianopsia\|snoring\|psychomotor agitation\|hypercapnia\|muscle hypertonia\|vertigo\|psychomotor disorders\|muscular atrophy\|consciousness disorders\|confusion\|dyslexia, acquired\|hearing loss, sensorineural\|vomiting\|apraxia, ideomotor\|hypokinesia\|vision disorders\|coma\|tremor\|obesity\|dyskinesias\|dysarthria\|paralysis\|hyperkinesis\|agraphia\|muscle weakness\|delirium\|anoxia\|catalepsy\|sleep disorders\|hypothermia\|deafness\|taste disorders\|body weight\|ataxia\|apraxias\|constipation\|seizures\|dyslexia\|unconsciousness\|gait ataxia\|amnesia\|myoclonus\|intermittent claudication\|headache\|alien hand syndrome\|speech disorders\|agnosia\|gait disorders, neurologic\|blindness\|cerebellar ataxia\|sensation disorders\|muscle rigidity\|amnesia, transient global\|hemiplegia\|ophthalmoplegia\|reflex, abnormal\|articulation disorders\|angina pectoris\|paraparesis, spastic\|paresis\|urinary incontinence, urge\|chorea\|pain, intractable\|mental fatigue\|fever\|pain\|overweight\|korsakoff syndrome\|fatigue\|dystonia\|communication disorders\|respiratory aspiration\|nocturia\|dizziness\|amnesia, anterograde\|vision, low\|perceptual disorders\|aphasia, wernicke\|urinary incontinence | | | |
| Asthma | Heart Failure | 0,519084 | sleep disorders\|edema, cardiac\|sleep deprivation\|angina pectoris\|edema\|abdominal pain\|fetal weight\|weight loss\|obesity\|psychophysiologic disorders\|hypothermia\|proteinuria\|body weight\|pain\|acute coronary syndrome\|hypercapnia\|spasm\|seizures\|vertigo\|taste disorders\|syncope\|paresthesia\|muscle hypotonia\|diarrhea\|hyperventilation\|thinness\|muscular atrophy\|myoclonus\|fetal hypoxia\|jaundice\|fetal distress\|memory disorders\|headache\|birth weight\|fever\|apnea\|respiratory sounds\|mental fatigue\|snoring\|asthenia\|overweight\|dyspnea, paroxysmal\|vomiting\|hiccup\|chest pain\|fatigue\|coma\|obesity, morbid\|cough\|hypoventilation\|weight gain\|nausea\|anorexia\|dyspepsia\|hemiplegia\|hypocapnia\|hemoptysis\|pruritus\|respiratory paralysis\|dyspnea\|muscle weakness\|mental retardation\|abdomen, acute\|dizziness\|anoxia\|reflex, abnormal\|cyanosis\|vocal cord paralysis | | | |
| Alzheimer Disease | Parkinson Disease | 0,5 | aphasia, broca\|sleep disorders\|mobility limitation\|hemiplegia\|sleep deprivation\|supranuclear palsy, progressive\|diarrhea\|primary progressive nonfluent aphasia\|body weight\|urinary incontinence\|articulation disorders\|weight loss\|echolalia\|psychophysiologic disorders\|hypothermia\|obesity\|urinary bladder, neurogenic\|catatonia\|ataxia\|snoring\|psychomotor agitation\|hypercapnia\|anomia\|seizures\|perceptual disorders\|taste disorders\|illusions\|syncope\|olfaction disorders\|fatigue\|urinary incontinence, urge\|apraxias\|chorea\|nocturia\|thinness\|muscular atrophy\|flushing\|agnosia\|anorexia\|coma\|memory disorders\|amnesia\|psychomotor disorders\|fever\|pain\|myoclonus\|communication disorders\|confusion\|learning disorders\|speech disorders\|vomiting\|apraxia, ideomotor\|dystonia\|aphasia\|hypokinesia\|vision disorders\|hallucinations\|tremor\|auditory perceptual disorders\|hyperphagia\|cerebellar ataxia\|weight gain\|nausea\|ophthalmoplegia, chronic progressive external\|gait disorders, neurologic\|akathisia, drug-induced\|dysarthria\|headache\|muscle rigidity\|paralysis\|color vision defects\|hyperkinesis\|agraphia\|urinary bladder, overactive\|muscle weakness\|mental retardation\|delirium\|dizziness\|language disorders\|anoxia\|reflex, abnormal\|gait apraxia | | | |
| Diabetes Mellitus, Type 2 | Obesity | 0,483221 | sleep disorders\|headache\|presbycusis\|albuminuria\|sleep deprivation\|hearing disorders\|edema\|abdominal pain\|pain, postoperative\|vision disorders\|urinary incontinence\|sarcopenia\|weight loss\|obesity\|psychophysiologic disorders\|cachexia\|proteinuria\|urinary bladder, neurogenic\|body weight\|snoring\|gastroparesis\|constipation\|seizures\|angina pectoris\|paresthesia\|virilism\|cardiac output, low\|diarrhea\|blindness\|nocturia\|thinness\|muscular atrophy\|deafness\|intermittent claudication\|polyuria\|obesity, morbid\|dyspnea\|memory disorders\|hirsutism\|birth weight\|pain\|anorexia\|bulimia\|learning disorders\|overweight\|flatulence\|hearing loss, sensorineural\|vomiting\|hyperalgesia\|fatigue\|prostatism\|aging, premature\|tremor\|hyperphagia\|dizziness\|nausea\|hypothermia\|gait disorders, neurologic\|dyspepsia\|fetal macrosomia\|quadriplegia\|acute coronary syndrome\|pruritus\|angina, unstable\|urinary bladder, overactive\|muscle weakness\|mental retardation\|vision, low\|weight gain\|urinary incontinence, stress\|anoxia\|taste disorders | | | |
| Hypertension | Migraine Disorders | 0,477833 | hearing disorders\|edema\|pain, postoperative\|weight loss\|hypothermia\|hyperesthesia\|syncope\|weight gain\|memory disorders\|tinnitus\|chest pain\|aphasia\|hypesthesia\|hallucinations\|nausea\|motion sickness\|obesity\|neck pain\|constipation\|hemianopsia\|abdominal pain\|eye manifestations\|muscle cramp\|psychomotor agitation\|hypercapnia\|spasm\|vertigo\|amaurosis fugax\|paresthesia\|flushing\|syncope, vasovagal\|consciousness disorders\|confusion\|bulimia\|asthenia\|hearing loss, sensorineural\|vomiting\|vision disorders\|coma\|tremor\|cough\|dizziness\|facial paralysis\|paralysis\|hyperkinesis\|hearing loss\|muscle weakness\|mental retardation\|anoxia\|sleep disorders\|sleep deprivation\|deafness\|abdomen, acute\|taste disorders\|body weight\|ataxia\|anorexia\|seizures\|unconsciousness\|neuralgia\|amnesia\|myoclonus\|intermittent claudication\|blindness, cortical\|psychophysiologic disorders\|headache\|ecchymosis\|learning disorders\|horner syndrome\|hyperalgesia\|blindness\|amnesia, transient global\|hemiplegia\|ophthalmoplegia\|akathisia, drug-induced\|reflex, abnormal\|angina pectoris\|hot flashes\|toothache\|postoperative nausea and vomiting\|paresis\|diarrhea\|chorea\|angina pectoris, variant\|colic\|pain, intractable\|mental fatigue\|fever\|pain\|back pain\|fatigue\|hypocapnia\|color vision defects\|hyperventilation\|dystonia\|facial pain\|scotoma | | | |
| Hypertension | Obesity | 0,465608 | edema, cardiac\|hearing disorders\|edema\|pain, postoperative\|weight loss\|urinary bladder, neurogenic\|hypothermia\|hyperoxia\|muscle hypotonia\|thinness\|weight gain\|tremor\|memory disorders\|apnea\|paraplegia\|nausea\|quadriplegia\|acute coronary syndrome\|urinary bladder, overactive\|obesity\|taste disorders\|albuminuria\|constipation\|cardiac output, low\|abdominal pain\|heartburn\|fever\|low back pain\|snoring\|hypercapnia\|vertigo\|psychomotor disorders\|paresthesia\|virilism\|presbycusis\|anorexia\|birth weight\|bulimia\|asthenia\|hearing loss, sensorineural\|vomiting\|prostatism\|vision disorders\|obesity, morbid\|dizziness\|fetal macrosomia\|hyperkinesis\|muscle weakness\|mental retardation\|delirium\|anoxia\|sleep disorders\|sleep deprivation\|deafness\|fetal weight\|proteinuria\|body weight\|dyspnea\|seizures\|pruritus\|hirsutism\|intermittent claudication\|psychophysiologic disorders\|headache\|ecchymosis\|learning disorders\|hyperalgesia\|blindness\|hyperphagia\|angina, unstable\|ageusia\|angina pectoris\|hot flashes\|postoperative nausea and vomiting\|diarrhea\|hyperventilation\|polyuria\|cheyne-stokes respiration\|hypercalciuria\|pain\|back pain\|overweight\|cyanosis\|fatigue\|hypoventilation\|arthralgia\|nocturia\|urinary incontinence | | | |
| Heart Failure | Hypertension | 0,460674 | sleep disorders\|edema, cardiac\|albuminuria\|sleep deprivation\|cardiac output, high\|edema\|abdominal pain\|fetal weight\|weight loss\|obesity\|psychophysiologic disorders\|hypothermia\|proteinuria\|body weight\|pain\|acute coronary syndrome\|hemianopsia\|hypercapnia\|constipation\|seizures\|fetal hypoxia\|vertigo\|hyperoxia\|angina pectoris\|syncope\|paresthesia\|muscle hypotonia\|cardiac output, low\|diarrhea\|hyperventilation\|fatigue\|nocturia\|thinness\|myoclonus\|deafness\|syncope, vasovagal\|spasm\|fetal distress\|memory disorders\|headache\|birth weight\|purpura, thrombotic thrombocytopenic\|hypercalciuria\|apnea\|fever\|tinnitus\|mental fatigue\|snoring\|confusion\|heart murmurs\|asthenia\|overweight\|dyspnea, paroxysmal\|vomiting\|intermittent claudication\|chest pain\|aphasia\|coma\|obesity, morbid\|oliguria\|cough\|hypoventilation\|weight gain\|nausea\|anorexia\|hypocapnia\|hemiplegia\|decerebrate state\|cheyne-stokes respiration\|angina pectoris, variant\|hemoptysis\|pruritus\|angina, unstable\|dyspnea\|muscle weakness\|mental retardation\|abdomen, acute\|dizziness\|anoxia\|reflex, abnormal\|cyanosis\|taste disorders | | | |
| Asthma | Myocardial Infarction | 0,457516 | sleep disorders\|neck pain\|edema, cardiac\|sleep deprivation\|angina pectoris\|edema\|abdominal pain\|pain, postoperative\|weight loss\|dyspnea\|hypergammaglobulinemia\|psychophysiologic disorders\|hypothermia\|proteinuria\|body weight\|pain\|hemoptysis\|hypercapnia\|spasm\|seizures\|vertigo\|snoring\|syncope\|unconsciousness\|paresthesia\|diarrhea\|hemiplegia\|hyperventilation\|thinness\|muscular atrophy\|flushing\|fetal hypoxia\|obesity, morbid\|jaundice\|heartburn\|memory disorders\|headache\|birth weight\|ecchymosis\|apnea\|respiratory sounds\|mental fatigue\|asthenia\|overweight\|dyspnea, paroxysmal\|paraplegia\|vomiting\|obesity\|chest pain\|fatigue\|coma\|tremor\|cough\|hypoventilation\|dizziness\|nausea\|dyspepsia\|paralysis\|quadriplegia\|hiccup\|fever\|acute coronary syndrome\|hyperkinesis\|pruritus\|cyanosis\|muscle weakness\|abdomen, acute\|weight gain\|anoxia\|reflex, abnormal | | | |
| Diabetes Mellitus, Type 2 | Hypertension | 0,455497 | hearing disorders\|edema\|pain, postoperative\|weight loss\|urinary bladder, neurogenic\|hypothermia\|thinness\|tremor\|memory disorders\|tinnitus\|chest pain\|hypesthesia\|hallucinations\|nausea\|quadriplegia\|acute coronary syndrome\|urinary bladder, overactive\|obesity\|taste disorders\|neck pain\|albuminuria\|constipation\|cardiac output, low\|abdominal pain\|muscle cramp\|snoring\|paresthesia\|virilism\|presbycusis\|flushing\|syncope, vasovagal\|birth weight\|confusion\|bulimia\|hearing loss, sensorineural\|vomiting\|prostatism\|vision disorders\|coma\|obesity, morbid\|cough\|weight gain\|anorexia\|facial paralysis\|fetal macrosomia\|hearing loss\|muscle weakness\|mental retardation\|anoxia\|sleep disorders\|sleep deprivation\|deafness\|proteinuria\|body weight\|dyspnea\|seizures\|pruritus\|hemianopsia\|hirsutism\|intermittent claudication\|psychophysiologic disorders\|headache\|learning disorders\|neuralgia\|hyperalgesia\|blindness\|hyperphagia\|hemiplegia\|ophthalmoplegia\|angina, unstable\|hearing loss, high-frequency\|reflex, abnormal\|angina pectoris\|hearing loss, sudden\|paresis\|diarrhea\|chorea\|polyuria\|pain\|overweight\|fatigue\|color vision defects\|nocturia\|dizziness\|dystonia\|scotoma\|urinary incontinence | | | |
| Heart Failure | Obesity | 0,439716 | sleep disorders\|mobility limitation\|edema, cardiac\|albuminuria\|sleep deprivation\|angina pectoris\|edema\|abdominal pain\|fetal weight\|weight loss\|obesity\|psychophysiologic disorders\|hypothermia\|proteinuria\|body weight\|pain\|snoring\|hypercapnia\|constipation\|seizures\|vertigo\|hyperoxia\|paresthesia\|muscle hypotonia\|cardiac output, low\|diarrhea\|hyperventilation\|nocturia\|thinness\|muscular atrophy\|deafness\|intermittent claudication\|jaundice\|memory disorders\|headache\|birth weight\|hypercalciuria\|apnea\|respiratory sounds\|asthenia\|overweight\|vomiting\|cyanosis\|fatigue\|obesity, morbid\|hypoventilation\|dizziness\|nausea\|anorexia\|dyspepsia\|cheyne-stokes respiration\|fever\|acute coronary syndrome\|pruritus\|angina, unstable\|dyspnea\|muscle weakness\|mental retardation\|cachexia\|weight gain\|anoxia\|taste disorders | | | |
| Asthma | Hypertension | 0,436842 | obesity, morbid\|sleep disorders\|neck pain\|edema, cardiac\|sleep deprivation\|angina pectoris\|edema\|abdominal pain\|pain, postoperative\|weight loss\|dyspnea\|hypergammaglobulinemia\|psychophysiologic disorders\|hypothermia\|proteinuria\|heartburn\|body weight\|pain\|hemoptysis\|hypercapnia\|muscle cramp\|diarrhea, infantile\|seizures\|hyperkinesis\|vertigo\|snoring\|syncope\|unconsciousness\|paresthesia\|hypoventilation\|muscle hypotonia\|neuralgia\|diarrhea\|hemiplegia\|tetany\|hyperventilation\|thinness\|hirsutism\|flushing\|fetal hypoxia\|fetal weight\|spasm\|blindness, cortical\|fetal distress\|memory disorders\|headache\|birth weight\|ecchymosis\|apnea\|myoclonus\|mental fatigue\|asthenia\|overweight\|dyspnea, paroxysmal\|paraplegia\|vomiting\|obesity\|chest pain\|fatigue\|akathisia, drug-induced\|coma\|tremor\|learning disorders\|cough\|low back pain\|weight gain\|nausea\|anorexia\|hypocapnia\|paralysis\|quadriplegia\|fever\|acute coronary syndrome\|purpura\|pruritus\|cyanosis\|muscle weakness\|mental retardation\|abdomen, acute\|dizziness\|anoxia\|reflex, abnormal\|taste disorders | | | |
| Hypertension | Stroke | 0,431718 | edema\|weight loss\|urinary bladder, neurogenic\|hyperoxia\|syncope\|muscle hypotonia\|scotoma\|tremor\|memory disorders\|apnea\|tinnitus\|paraplegia\|chest pain\|aphasia\|hypesthesia\|hallucinations\|nausea\|quadriplegia\|acute coronary syndrome\|urinary bladder, overactive\|weight gain\|taste disorders\|neck pain\|albuminuria\|constipation\|hemianopsia\|snoring\|psychomotor agitation\|hypercapnia\|spasm\|muscle hypertonia\|vertigo\|psychomotor disorders\|amaurosis fugax\|paresthesia\|birth weight\|consciousness disorders\|confusion\|hearing loss, sensorineural\|vomiting\|vision disorders\|coma\|obesity, morbid\|cough\|obesity\|facial paralysis\|paralysis\|hyperkinesis\|muscle weakness\|delirium\|anoxia\|catalepsy\|sleep disorders\|hypothermia\|deafness\|proteinuria\|body weight\|ataxia\|dyspnea\|seizures\|unconsciousness\|cardiac output, low\|amnesia\|myoclonus\|intermittent claudication\|blindness, cortical\|headache\|neuralgia\|horner syndrome\|hyperalgesia\|blindness\|muscle rigidity\|amnesia, transient global\|hemiplegia\|ophthalmoplegia\|angina, unstable\|dysgeusia\|reflex, abnormal\|angina pectoris\|hot flashes\|hearing loss, sudden\|paresis\|chorea\|hyperventilation\|pain, intractable\|mental fatigue\|cheyne-stokes respiration\|fever\|pain\|overweight\|fatigue\|arthralgia\|purpura, thrombotic thrombocytopenic\|nocturia\|dizziness\|dystonia\|facial pain\|urinary incontinence | | | |
| Migraine Disorders | Parkinson Disease | 0,421875 | aphasia, broca\|sleep disorders\|headache\|dysarthria\|sleep deprivation\|edema\|eye manifestations\|weight loss\|reflex, babinski\|coma\|psychophysiologic disorders\|hypothermia\|obesity\|muscle cramp\|body weight\|ataxia\|color vision defects\|psychomotor agitation\|hypercapnia\|constipation\|seizures\|perceptual disorders\|somatosensory disorders\|vertigo\|illusions\|syncope\|paresthesia\|gastroparesis\|paresis\|torticollis\|diarrhea\|hemiplegia\|facial pain\|chorea\|shoulder pain\|flushing\|phantom limb\|spasm\|pain, intractable\|memory disorders\|amnesia\|sensation disorders\|learning disorders\|pain\|myoclonus\|mental fatigue\|confusion\|back pain\|asthenia\|speech disorders\|vomiting\|hyperalgesia\|dystonia\|aphasia\|vision disorders\|hallucinations\|tremor\|cough\|cerebellar ataxia\|dizziness\|nausea\|anorexia\|facial paralysis\|olfaction disorders\|dyspepsia\|diplopia\|paralysis\|ophthalmoplegia\|fever\|hyperkinesis\|agraphia\|fatigue\|hyperventilation\|muscle weakness\|mental retardation\|akathisia, drug-induced\|weight gain\|language disorders\|anoxia\|reflex, abnormal\|taste disorders | | | |
| Alzheimer Disease | Stroke | 0,419355 | aphasia, broca\|sleep disorders\|mobility limitation\|hemiplegia\|anomia\|agnosia\|hemianopsia\|body weight\|urinary incontinence\|articulation disorders\|weight loss\|coma\|obesity\|urinary bladder, neurogenic\|tremor\|ataxia\|paraparesis, spastic\|snoring\|psychomotor agitation\|hypercapnia\|hypothermia\|seizures\|perceptual disorders\|aphasia, primary progressive\|illusions\|syncope\|unconsciousness\|fatigue\|urinary incontinence, urge\|apraxias\|mutism\|chorea\|nocturia\|muscular atrophy\|myoclonus\|confusion\|memory disorders\|amnesia\|psychomotor disorders\|fever\|pain\|dyslexia\|communication disorders\|persistent vegetative state\|dyslexia, acquired\|speech disorders\|vomiting\|prosopagnosia\|korsakoff syndrome\|apraxia, ideomotor\|dystonia\|aphasia\|hypokinesia\|vision disorders\|hallucinations\|blindness\|auditory perceptual disorders\|cerebellar ataxia\|weight gain\|nausea\|gait disorders, neurologic\|language development disorders\|dysarthria\|headache\|muscle rigidity\|amnesia, transient global\|paralysis\|hyperkinesis\|agraphia\|urinary bladder, overactive\|muscle weakness\|delirium\|dizziness\|language disorders\|anoxia\|reflex, abnormal\|aphasia, wernicke\|taste disorders | | | |
| Migraine Disorders | Stroke | 0,412621 | aphasia, broca\|sleep disorders\|neck pain\|dysarthria\|constipation\|angina pectoris\|edema\|hot flashes\|deafness\|weight loss\|reflex, babinski\|coma\|fever\|hypothermia\|obesity\|body weight\|ataxia\|dystonia\|psychomotor agitation\|hypercapnia\|spasm\|seizures\|perceptual disorders\|somatosensory disorders\|vertigo\|illusions\|syncope\|unconsciousness\|paresthesia\|paresis\|neuralgia\|blindness\|chorea\|shoulder pain\|myoclonus\|scotoma\|intermittent claudication\|phantom limb\|pain, intractable\|blindness, cortical\|memory disorders\|amnesia\|consciousness disorders\|pain\|tinnitus\|mental fatigue\|confusion\|dyslexia, acquired\|speech disorders\|hearing loss, sensorineural\|vomiting\|horner syndrome\|hyperalgesia\|chest pain\|aphasia\|hypesthesia\|vision disorders\|hallucinations\|tremor\|cough\|cerebellar ataxia\|dizziness\|nausea\|sensation disorders\|facial paralysis\|facial pain\|amaurosis fugax\|diplopia\|hemiplegia\|amnesia, transient global\|hemianopsia\|paralysis\|headache\|hyperkinesis\|agraphia\|vision, low\|hyperventilation\|muscle weakness\|ophthalmoplegia\|fatigue\|weight gain\|language disorders\|anoxia\|reflex, abnormal\|taste disorders | | | |
| Dementia | Migraine Disorders | 0,411168 | aphasia, broca\|sleep disorders\|hemiplegia\|sleep deprivation\|hearing disorders\|hemianopsia\|agraphia\|deafness\|eye manifestations\|weight loss\|color vision defects\|coma\|psychophysiologic disorders\|hypothermia\|obesity\|body weight\|ataxia\|psychomotor agitation\|hypercapnia\|constipation\|seizures\|perceptual disorders\|vertigo\|illusions\|syncope\|unconsciousness\|olfaction disorders\|paresis\|torticollis\|diarrhea\|blindness\|chorea\|amnesia\|flushing\|intermittent claudication\|syncope, vasovagal\|pain, intractable\|pain, postoperative\|memory disorders\|headache\|sensation disorders\|consciousness disorders\|pain\|myoclonus\|tinnitus\|mental fatigue\|confusion\|dyslexia, acquired\|speech disorders\|hearing loss, sensorineural\|vomiting\|dystonia\|aphasia\|asthenia\|vision disorders\|hallucinations\|tremor\|learning disorders\|cerebellar ataxia\|dizziness\|nausea\|anorexia\|dysarthria\|amnesia, transient global\|paralysis\|amnesia, retrograde\|ophthalmoplegia\|fever\|vision, low\|hyperkinesis\|hearing loss\|fatigue\|muscle weakness\|mental retardation\|angina pectoris\|akathisia, drug-induced\|weight gain\|language disorders\|anoxia\|reflex, abnormal\|taste disorders | | | |
| Myocardial Infarction | Obesity | 0,401235 | sleep disorders\|edema, cardiac\|albuminuria\|sleep deprivation\|angina pectoris\|edema\|abdominal pain\|pain, postoperative\|weight loss\|dyspnea\|psychophysiologic disorders\|cachexia\|proteinuria\|body weight\|pain\|snoring\|gastroparesis\|hypercapnia\|constipation\|seizures\|vertigo\|hyperoxia\|psychomotor disorders\|paresthesia\|cardiac output, low\|diarrhea\|blindness\|hyperventilation\|thinness\|muscular atrophy\|intermittent claudication\|obesity, morbid\|jaundice\|heartburn\|memory disorders\|headache\|birth weight\|ecchymosis\|apnea\|respiratory sounds\|back pain\|asthenia\|overweight\|paraplegia\|vomiting\|obesity\|cyanosis\|fatigue\|tremor\|hypoventilation\|dizziness\|nausea\|hypothermia\|dyspepsia\|quadriplegia\|fever\|acute coronary syndrome\|hyperkinesis\|pruritus\|angina, unstable\|urinary bladder, overactive\|muscle weakness\|delirium\|weight gain\|anoxia | | | |
| Diabetes Mellitus, Type 2 | Migraine Disorders | 0,394286 | sleep disorders\|neck pain\|olfaction disorders\|sleep deprivation\|hearing disorders\|edema\|abdominal pain\|pain, postoperative\|earache\|weight loss\|color vision defects\|coma\|psychophysiologic disorders\|hypothermia\|taste disorders\|muscle cramp\|body weight\|gastroparesis\|constipation\|seizures\|angina pectoris\|paresthesia\|paresis\|hemianopsia\|diarrhea\|blindness\|chorea\|shoulder pain\|flushing\|deafness\|intermittent claudication\|syncope, vasovagal\|memory disorders\|headache\|sensation disorders\|pain\|tinnitus\|confusion\|bulimia\|learning disorders\|neuralgia\|hearing loss, sensorineural\|vomiting\|hyperalgesia\|chest pain\|fatigue\|hypesthesia\|vision disorders\|hallucinations\|tremor\|cough\|obesity\|nausea\|anorexia\|facial paralysis\|weight gain\|dyspepsia\|diplopia\|hemiplegia\|ophthalmoplegia\|hearing loss\|vision, low\|muscle weakness\|mental retardation\|dystonia\|dizziness\|anoxia\|reflex, abnormal\|scotoma | | | |
| Asthma | Obesity | 0,393548 | sleep disorders\|edema, cardiac\|sleep deprivation\|angina pectoris\|edema\|abdominal pain\|heartburn\|weight loss\|respiratory aspiration\|obesity\|psychophysiologic disorders\|hypothermia\|proteinuria\|body weight\|pain\|snoring\|hypercapnia\|dyspnea\|seizures\|obesity, morbid\|vertigo\|paresthesia\|hypoventilation\|muscle hypotonia\|diarrhea\|thinness\|muscular atrophy\|fetal weight\|jaundice\|pain, postoperative\|memory disorders\|hirsutism\|birth weight\|ecchymosis\|apnea\|respiratory sounds\|asthenia\|overweight\|paraplegia\|vomiting\|cyanosis\|fatigue\|tremor\|learning disorders\|low back pain\|weight gain\|nausea\|anorexia\|dyspepsia\|quadriplegia\|fever\|acute coronary syndrome\|hyperkinesis\|pruritus\|hyperventilation\|muscle weakness\|mental retardation\|dizziness\|headache\|anoxia\|taste disorders | | | |
| Dementia | Hypertension | 0,386667 | hearing disorders\|pain, postoperative\|weight loss\|urinary bladder, neurogenic\|hypothermia\|syncope\|thinness\|memory disorders\|tinnitus\|paraplegia\|aphasia\|hallucinations\|nausea\|quadriplegia\|urinary bladder, overactive\|weight gain\|constipation\|supranuclear palsy, progressive\|hemianopsia\|eye manifestations\|low back pain\|snoring\|psychomotor agitation\|hypercapnia\|muscle hypertonia\|vertigo\|psychomotor disorders\|presbycusis\|flushing\|syncope, vasovagal\|consciousness disorders\|confusion\|asthenia\|hearing loss, sensorineural\|vomiting\|vision disorders\|coma\|tremor\|dizziness\|paralysis\|hyperkinesis\|hearing loss\|muscle weakness\|mental retardation\|delirium\|anoxia\|catalepsy\|sleep disorders\|sleep deprivation\|deafness\|taste disorders\|body weight\|ataxia\|anorexia\|seizures\|unconsciousness\|amnesia\|myoclonus\|intermittent claudication\|psychophysiologic disorders\|headache\|learning disorders\|blindness\|hyperphagia\|muscle rigidity\|amnesia, transient global\|hemiplegia\|ophthalmoplegia\|akathisia, drug-induced\|reflex, abnormal\|angina pectoris\|obesity\|paresis\|diarrhea\|chorea\|polyuria\|pain, intractable\|mental fatigue\|fever\|pain\|overweight\|hypergammaglobulinemia\|fatigue\|color vision defects\|nocturia\|dystonia\|urinary incontinence | | | |
| Diabetes Mellitus, Type 2 | Myocardial Infarction | 0,381818 | sleep disorders\|neck pain\|albuminuria\|sleep deprivation\|angina pectoris\|edema\|abdominal pain\|pain, postoperative\|body weight\|cardiac output, low\|weight loss\|coma\|psychophysiologic disorders\|cachexia\|proteinuria\|obesity, morbid\|snoring\|gastroparesis\|constipation\|seizures\|paresthesia\|paresis\|hemianopsia\|diarrhea\|blindness\|thinness\|muscular atrophy\|flushing\|syncope, vasovagal\|dyspnea\|memory disorders\|headache\|birth weight\|pain\|confusion\|overweight\|vomiting\|intermittent claudication\|chest pain\|fatigue\|hypesthesia\|hemoglobinuria\|hallucinations\|tremor\|cough\|obesity\|nausea\|hypothermia\|facial paralysis\|weight gain\|dyskinesias\|dyspepsia\|hemiplegia\|quadriplegia\|acute coronary syndrome\|pruritus\|angina, unstable\|urinary bladder, overactive\|muscle weakness\|hearing loss, sudden\|dizziness\|anoxia\|reflex, abnormal | | | |
| Myocardial Infarction | Stroke | 0,375 | sleep disorders\|neck pain\|hemiplegia\|albuminuria\|constipation\|angina pectoris\|edema\|cardiac output, low\|weight loss\|coma\|hypothermia\|obesity\|body weight\|pain\|snoring\|psychomotor agitation\|hypercapnia\|spasm\|seizures\|vertigo\|hyperoxia\|psychomotor disorders\|syncope\|unconsciousness\|paresthesia\|paresis\|hemianopsia\|fatigue\|blindness\|hyperventilation\|hearing loss, sudden\|reflex, abnormal\|muscular atrophy\|confusion\|intermittent claudication\|obesity, morbid\|dyspnea\|memory disorders\|headache\|birth weight\|fever\|apnea\|mental fatigue\|purpura, thrombocytopenic, idiopathic\|dyslexia, acquired\|overweight\|paraplegia\|vomiting\|hiccup\|chest pain\|aphasia\|hypesthesia\|hallucinations\|tremor\|cough\|purpura, thrombotic thrombocytopenic\|dizziness\|nausea\|facial paralysis\|dyskinesias\|muscle rigidity\|amnesia, transient global\|paralysis\|quadriplegia\|acute coronary syndrome\|hyperkinesis\|angina, unstable\|urinary bladder, overactive\|muscle weakness\|delirium\|weight gain\|facial pain\|anoxia\|catalepsy\|proteinuria | | | |
| Migraine Disorders | Myocardial Infarction | 0,373626 | sleep disorders\|neck pain\|hemiplegia\|sleep deprivation\|angina pectoris\|edema\|abdominal pain\|eye manifestations\|weight loss\|coma\|psychophysiologic disorders\|hypothermia\|obesity\|body weight\|ecchymosis\|psychomotor agitation\|hypercapnia\|spasm\|seizures\|vertigo\|syncope\|unconsciousness\|paresthesia\|gastroparesis\|paresis\|hemianopsia\|diarrhea\|blindness\|constipation\|flushing\|intermittent claudication\|syncope, vasovagal\|pain, postoperative\|memory disorders\|headache\|dyslexia, acquired\|pain\|mental fatigue\|confusion\|back pain\|asthenia\|pain, referred\|vomiting\|angina pectoris, variant\|chest pain\|aphasia\|hypesthesia\|hallucinations\|tremor\|cough\|dizziness\|nausea\|facial paralysis\|dyspepsia\|amnesia, transient global\|paralysis\|colic\|fever\|hyperkinesis\|fatigue\|hyperventilation\|muscle weakness\|abdomen, acute\|weight gain\|facial pain\|anoxia\|reflex, abnormal\|toothache | | | |
| Hypertension | Parkinson Disease | 0,370536 | sleep disorders\|hemiplegia\|sleep deprivation\|supranuclear palsy, progressive\|edema\|eye manifestations\|weight loss\|color vision defects\|coma\|psychophysiologic disorders\|hypothermia\|taste disorders\|muscle cramp\|body weight\|ataxia\|snoring\|psychomotor agitation\|hypercapnia\|spasm\|seizures\|muscle hypertonia\|vertigo\|akathisia, drug-induced\|psychomotor disorders\|syncope\|hemifacial spasm\|paresthesia\|muscle hypotonia\|fatigue\|diarrhea\|hyperventilation\|constipation\|chorea\|reflex, abnormal\|thinness\|amnesia\|flushing\|dyspnea\|pain, intractable\|dizziness\|memory disorders\|headache\|cheyne-stokes respiration\|learning disorders\|pain\|myoclonus\|mental fatigue\|confusion\|back pain\|asthenia\|paresis\|paraplegia\|vomiting\|hyperalgesia\|dystonia\|aphasia\|vision disorders\|hallucinations\|tremor\|hyperphagia\|urinary bladder, neurogenic\|hypoventilation\|weight gain\|nausea\|anorexia\|facial paralysis\|muscle rigidity\|paralysis\|quadriplegia\|ophthalmoplegia\|fever\|nocturia\|hyperkinesis\|cough\|urinary bladder, overactive\|muscle weakness\|mental retardation\|delirium\|obesity\|facial pain\|anoxia\|catalepsy\|urinary incontinence | | | |
| Dementia | Diabetes Mellitus, Type 2 | 0,361702 | sleep disorders\|olfaction disorders\|sleep deprivation\|hearing disorders\|hemianopsia\|deafness\|pain, postoperative\|vision disorders\|weight loss\|color vision defects\|coma\|psychophysiologic disorders\|cachexia\|taste disorders\|urinary bladder, neurogenic\|body weight\|snoring\|constipation\|seizures\|angina pectoris\|paresis\|presbycusis\|diarrhea\|blindness\|ophthalmoplegia, chronic progressive external\|chorea\|nocturia\|thinness\|muscular atrophy\|flushing\|intermittent claudication\|polyuria\|syncope, vasovagal\|memory disorders\|headache\|sensation disorders\|pain\|tinnitus\|confusion\|learning disorders\|overweight\|hearing loss, sensorineural\|vomiting\|fatigue\|gait disorders, neurologic\|hallucinations\|tremor\|hyperphagia\|obesity\|nausea\|hypothermia\|weight gain\|dyskinesias\|vision, low\|hemiplegia\|quadriplegia\|ophthalmoplegia\|anorexia\|hearing loss\|aging, premature\|urinary bladder, overactive\|muscle weakness\|mental retardation\|dystonia\|dizziness\|anoxia\|reflex, abnormal\|urinary incontinence | | | |
| Diabetes Mellitus, Type 2 | Heart Failure | 0,36 | sleep disorders\|albuminuria\|sleep deprivation\|angina pectoris\|edema\|abdominal pain\|weight loss\|obesity\|psychophysiologic disorders\|hypothermia\|proteinuria\|body weight\|snoring\|hemianopsia\|constipation\|seizures\|dyspnea\|paresthesia\|cardiac output, low\|diarrhea\|thinness\|muscular atrophy\|deafness\|intermittent claudication\|syncope, vasovagal\|memory disorders\|headache\|birth weight\|pain\|tinnitus\|confusion\|overweight\|vomiting\|chest pain\|fatigue\|coma\|obesity, morbid\|cough\|dizziness\|nausea\|anorexia\|dyspepsia\|hemiplegia\|acute coronary syndrome\|pruritus\|angina, unstable\|nocturia\|muscle weakness\|mental retardation\|cachexia\|weight gain\|anoxia\|reflex, abnormal\|taste disorders | | | |
| Alzheimer Disease | Migraine Disorders | 0,357955 | aphasia, broca\|sleep disorders\|hemiplegia\|sleep deprivation\|hearing disorders\|hemianopsia\|agraphia\|body weight\|weight loss\|coma\|psychophysiologic disorders\|hypothermia\|obesity\|tremor\|ataxia\|psychomotor agitation\|hypercapnia\|syncope, vasovagal\|seizures\|perceptual disorders\|illusions\|syncope\|unconsciousness\|olfaction disorders\|fatigue\|diarrhea\|chorea\|amnesia\|flushing\|anorexia\|memory disorders\|headache\|learning disorders\|pain\|myoclonus\|confusion\|dyslexia, acquired\|speech disorders\|vomiting\|dystonia\|aphasia\|vision disorders\|hallucinations\|blindness\|cerebellar ataxia\|dizziness\|nausea\|dysarthria\|amnesia, transient global\|paralysis\|amnesia, retrograde\|color vision defects\|fever\|hyperkinesis\|hearing loss\|muscle weakness\|mental retardation\|akathisia, drug-induced\|weight gain\|language disorders\|anoxia\|reflex, abnormal\|taste disorders | | | |
| Diabetes Mellitus, Type 2 | Stroke | 0,351759 | sleep disorders\|neck pain\|vision, low\|albuminuria\|constipation\|angina pectoris\|edema\|deafness\|vision disorders\|cardiac output, low\|weight loss\|body weight\|coma\|hypothermia\|proteinuria\|urinary bladder, neurogenic\|obesity, morbid\|dystonia\|snoring\|overweight\|dyspnea\|seizures\|urinary incontinence\|paresthesia\|paresis\|hemianopsia\|blindness\|pupil disorders\|chorea\|nocturia\|shoulder pain\|scotoma\|intermittent claudication\|memory disorders\|muscular atrophy\|birth weight\|pain\|tinnitus\|confusion\|neuralgia\|hearing loss, sensorineural\|vomiting\|hyperalgesia\|chest pain\|fatigue\|hypesthesia\|gait disorders, neurologic\|hallucinations\|tremor\|cough\|obesity\|nausea\|sensation disorders\|facial paralysis\|weight gain\|dyskinesias\|diplopia\|hemiplegia\|quadriplegia\|ophthalmoplegia\|acute coronary syndrome\|angina, unstable\|urinary bladder, overactive\|muscle weakness\|hearing loss, sudden\|dizziness\|headache\|anoxia\|reflex, abnormal\|taste disorders | | | |
| Asthma | Parkinson Disease | 0,351648 | sleep disorders\|hemiplegia\|sleep deprivation\|edema\|diarrhea\|weight loss\|psychophysiologic disorders\|hypothermia\|obesity\|muscle cramp\|body weight\|respiratory aspiration\|snoring\|hypercapnia\|dyspnea\|seizures\|vertigo\|syncope\|paresthesia\|hypoventilation\|muscle hypotonia\|torticollis\|dysphonia\|hoarseness\|thinness\|muscular atrophy\|flushing\|spasm\|memory disorders\|headache\|learning disorders\|pain\|myoclonus\|respiratory sounds\|mental fatigue\|asthenia\|gagging\|paraplegia\|vomiting\|apraxia, ideomotor\|fatigue\|coma\|tremor\|fever\|cough\|olfaction disorders\|dizziness\|nausea\|anorexia\|dyspepsia\|paralysis\|quadriplegia\|hiccup\|voice disorders\|vocal cord paralysis\|hyperkinesis\|hyperventilation\|muscle weakness\|mental retardation\|akathisia, drug-induced\|weight gain\|anoxia\|reflex, abnormal\|taste disorders | | | |
| Diabetes Mellitus, Type 2 | Parkinson Disease | 0,342246 | sleep disorders\|headache\|olfaction disorders\|sleep deprivation\|edema\|vision disorders\|weight loss\|color vision defects\|coma\|psychophysiologic disorders\|hypothermia\|taste disorders\|muscle cramp\|body weight\|snoring\|gastroparesis\|constipation\|seizures\|hyperphagia\|paresthesia\|urinary bladder, neurogenic\|diarrhea\|ophthalmoplegia, chronic progressive external\|pupil disorders\|chorea\|nocturia\|thinness\|shoulder pain\|flushing\|dyspnea\|memory disorders\|muscular atrophy\|sensation disorders\|pain\|confusion\|learning disorders\|vomiting\|hyperalgesia\|dystonia\|fatigue\|gait disorders, neurologic\|hallucinations\|tremor\|cough\|obesity\|nausea\|anorexia\|facial paralysis\|weight gain\|dyskinesias\|dyspepsia\|diplopia\|hemiplegia\|quadriplegia\|ophthalmoplegia\|urinary bladder, overactive\|muscle weakness\|mental retardation\|paresis\|dizziness\|urinary incontinence, stress\|anoxia\|reflex, abnormal\|urinary incontinence | | | |
| Myocardial Infarction | Parkinson Disease | 0,338542 | sleep disorders\|gastroparesis\|hemiplegia\|sleep deprivation\|edema\|eye manifestations\|weight loss\|coma\|psychophysiologic disorders\|hypothermia\|obesity\|body weight\|snoring\|psychomotor agitation\|hypercapnia\|spasm\|seizures\|vertigo\|psychomotor disorders\|syncope\|paresthesia\|eructation\|paresis\|fatigue\|diarrhea\|hyperventilation\|constipation\|reflex, abnormal\|thinness\|muscular atrophy\|flushing\|dyspnea\|memory disorders\|headache\|fever\|pain\|respiratory sounds\|mental fatigue\|confusion\|back pain\|asthenia\|paraplegia\|vomiting\|hiccup\|aphasia\|hallucinations\|tremor\|cough\|hypoventilation\|dizziness\|nausea\|facial paralysis\|dyskinesias\|dyspepsia\|muscle rigidity\|paralysis\|quadriplegia\|hyperkinesis\|urinary bladder, overactive\|muscle weakness\|delirium\|weight gain\|facial pain\|anoxia\|catalepsy | | | |
| Asthma | Migraine Disorders | 0,335196 | sleep disorders\|neck pain\|hemiplegia\|sleep deprivation\|angina pectoris\|edema\|abdominal pain\|pain, postoperative\|weight loss\|psychophysiologic disorders\|hypothermia\|obesity\|muscle cramp\|body weight\|sciatica\|hypercapnia\|spasm\|seizures\|vertigo\|syncope\|unconsciousness\|paresthesia\|neuralgia\|diarrhea\|dysmenorrhea\|flushing\|anorexia\|blindness, cortical\|memory disorders\|headache\|ecchymosis\|pain\|myoclonus\|mental fatigue\|asthenia\|torticollis\|vomiting\|chest pain\|fatigue\|akathisia, drug-induced\|coma\|tremor\|learning disorders\|cough\|olfaction disorders\|dizziness\|nausea\|dyspepsia\|paralysis\|hypocapnia\|fever\|hyperkinesis\|hyperventilation\|muscle weakness\|mental retardation\|abdomen, acute\|weight gain\|anoxia\|reflex, abnormal\|taste disorders | | | |
| Dementia | Obesity | 0,335079 | sleep disorders\|mobility limitation\|vision, low\|sleep deprivation\|hearing disorders\|deafness\|pain, postoperative\|vision disorders\|weight loss\|psychophysiologic disorders\|cachexia\|obesity\|urinary bladder, neurogenic\|body weight\|low back pain\|snoring\|hypercapnia\|constipation\|seizures\|perceptual disorders\|vertigo\|psychomotor disorders\|presbycusis\|urinary incontinence, urge\|blindness\|nocturia\|thinness\|muscular atrophy\|intermittent claudication\|polyuria\|jaundice\|memory disorders\|headache\|learning disorders\|pain\|respiratory aspiration\|asthenia\|overweight\|paraplegia\|vomiting\|fatigue\|hearing loss, sensorineural\|aging, premature\|tremor\|hyperphagia\|dizziness\|nausea\|hypothermia\|gait disorders, neurologic\|language development disorders\|quadriplegia\|fever\|anorexia\|hyperkinesis\|urinary bladder, overactive\|muscle weakness\|mental retardation\|angina pectoris\|delirium\|weight gain\|urinary incontinence\|anoxia\|diarrhea\|taste disorders | | | |
| Obesity | Parkinson Disease | 0,322751 | sleep disorders\|mobility limitation\|sleep deprivation\|edema\|diarrhea\|gait disorders, neurologic\|urinary incontinence\|weight loss\|psychophysiologic disorders\|hypothermia\|obesity\|urinary bladder, neurogenic\|body weight\|respiratory aspiration\|snoring\|gastroparesis\|hypercapnia\|constipation\|seizures\|perceptual disorders\|vertigo\|psychomotor disorders\|paresthesia\|muscle hypotonia\|urinary incontinence, urge\|hyperventilation\|nocturia\|thinness\|muscular atrophy\|dyspnea\|memory disorders\|headache\|cheyne-stokes respiration\|learning disorders\|pain\|respiratory sounds\|back pain\|asthenia\|paraplegia\|vomiting\|hyperalgesia\|fatigue\|vision disorders\|tremor\|hyperphagia\|hypoventilation\|dizziness\|nausea\|anorexia\|dyspepsia\|quadriplegia\|fever\|hyperkinesis\|urinary bladder, overactive\|muscle weakness\|mental retardation\|delirium\|weight gain\|urinary incontinence, stress\|anoxia\|taste disorders | | | |
| Heart Failure | Stroke | 0,321053 | sleep disorders\|mobility limitation\|albuminuria\|constipation\|angina pectoris\|edema\|deafness\|weight loss\|obesity\|hypothermia\|proteinuria\|body weight\|pain\|vocal cord paralysis\|hemianopsia\|hypercapnia\|spasm\|seizures\|vertigo\|hyperoxia\|syncope\|paresthesia\|muscle hypotonia\|cardiac output, low\|angina, unstable\|hyperventilation\|muscular atrophy\|myoclonus\|intermittent claudication\|dyspnea\|memory disorders\|headache\|birth weight\|fever\|apnea\|tinnitus\|mental fatigue\|snoring\|confusion\|overweight\|vomiting\|hiccup\|chest pain\|aphasia\|coma\|obesity, morbid\|cough\|purpura, thrombotic thrombocytopenic\|dizziness\|nausea\|hemiplegia\|cheyne-stokes respiration\|acute coronary syndrome\|respiratory paralysis\|nocturia\|muscle weakness\|fatigue\|weight gain\|anoxia\|reflex, abnormal\|taste disorders | | | |
| Obesity | Stroke | 0,320197 | sleep disorders\|mobility limitation\|vision, low\|albuminuria\|constipation\|angina pectoris\|edema\|hot flashes\|deafness\|gait disorders, neurologic\|weight loss\|obesity\|hypothermia\|proteinuria\|urinary bladder, neurogenic\|body weight\|pain\|snoring\|hypercapnia\|dyspnea\|seizures\|perceptual disorders\|vertigo\|hyperoxia\|psychomotor disorders\|paresthesia\|muscle hypotonia\|cardiac output, low\|urinary incontinence, urge\|blindness\|hyperventilation\|nocturia\|muscular atrophy\|intermittent claudication\|obesity, morbid\|memory disorders\|headache\|birth weight\|fever\|apnea\|overweight\|paraplegia\|vomiting\|hyperalgesia\|fatigue\|hearing loss, sensorineural\|vision disorders\|tremor\|dizziness\|nausea\|language development disorders\|arthralgia\|quadriplegia\|cheyne-stokes respiration\|respiratory aspiration\|acute coronary syndrome\|hyperkinesis\|angina, unstable\|urinary bladder, overactive\|muscle weakness\|delirium\|weight gain\|urinary incontinence\|anoxia\|taste disorders | | | |
| Dementia | Myocardial Infarction | 0,316583 | sleep disorders\|hemiplegia\|sleep deprivation\|angina pectoris\|hemianopsia\|eye manifestations\|weight loss\|coma\|psychophysiologic disorders\|cachexia\|obesity\|body weight\|snoring\|psychomotor agitation\|hypercapnia\|constipation\|seizures\|vertigo\|psychomotor disorders\|syncope\|unconsciousness\|paresis\|fatigue\|diarrhea\|blindness\|reflex, abnormal\|thinness\|muscular atrophy\|flushing\|syncope, vasovagal\|jaundice\|pain, postoperative\|memory disorders\|headache\|dyslexia, acquired\|pain\|mental fatigue\|confusion\|asthenia\|overweight\|paraplegia\|vomiting\|intermittent claudication\|hypergammaglobulinemia\|aphasia\|hallucinations\|tremor\|dizziness\|nausea\|hypothermia\|dyskinesias\|muscle rigidity\|amnesia, transient global\|paralysis\|quadriplegia\|fever\|hyperkinesis\|urinary bladder, overactive\|muscle weakness\|delirium\|weight gain\|anoxia\|catalepsy | | | |
| Asthma | Diabetes Mellitus, Type 2 | 0,315152 | sleep disorders\|neck pain\|olfaction disorders\|sleep deprivation\|angina pectoris\|edema\|abdominal pain\|pain, postoperative\|weight loss\|obesity\|psychophysiologic disorders\|hypothermia\|proteinuria\|muscle cramp\|body weight\|snoring\|dyspnea\|seizures\|paresthesia\|neuralgia\|diarrhea\|thinness\|muscular atrophy\|flushing\|obesity, morbid\|anorexia\|memory disorders\|hirsutism\|birth weight\|pain\|learning disorders\|overweight\|vomiting\|chest pain\|fatigue\|coma\|tremor\|cough\|dizziness\|nausea\|dyspepsia\|hemiplegia\|quadriplegia\|acute coronary syndrome\|pruritus\|muscle weakness\|mental retardation\|weight gain\|headache\|anoxia\|reflex, abnormal\|taste disorders | | | |
| Alzheimer Disease | Diabetes Mellitus, Type 2 | 0,315152 | sleep disorders\|olfaction disorders\|sleep deprivation\|hearing disorders\|hemianopsia\|vision disorders\|weight loss\|body weight\|coma\|psychophysiologic disorders\|cachexia\|obesity\|urinary bladder, neurogenic\|tremor\|snoring\|syncope, vasovagal\|seizures\|diarrhea\|ophthalmoplegia, chronic progressive external\|chorea\|nocturia\|thinness\|muscular atrophy\|flushing\|anorexia\|memory disorders\|headache\|pain\|confusion\|learning disorders\|vomiting\|fatigue\|aging, premature\|hallucinations\|blindness\|hyperphagia\|dizziness\|nausea\|hypothermia\|gait disorders, neurologic\|hemiplegia\|color vision defects\|hearing loss\|urinary bladder, overactive\|muscle weakness\|mental retardation\|dystonia\|weight gain\|urinary incontinence\|anoxia\|reflex, abnormal\|taste disorders | | | |
| Heart Failure | Migraine Disorders | 0,306358 | sleep disorders\|sleep deprivation\|angina pectoris\|edema\|abdominal pain\|weight loss\|psychophysiologic disorders\|hypothermia\|obesity\|body weight\|hypercapnia\|spasm\|seizures\|vertigo\|syncope\|paresthesia\|hemianopsia\|diarrhea\|constipation\|myoclonus\|deafness\|intermittent claudication\|syncope, vasovagal\|memory disorders\|headache\|fever\|pain\|tinnitus\|mental fatigue\|confusion\|asthenia\|vomiting\|angina pectoris, variant\|chest pain\|aphasia\|coma\|cough\|dizziness\|nausea\|anorexia\|dyspepsia\|hemiplegia\|hypocapnia\|fatigue\|hyperventilation\|muscle weakness\|mental retardation\|abdomen, acute\|weight gain\|neuralgia, postherpetic\|anoxia\|reflex, abnormal\|taste disorders | | | |
| Alzheimer Disease | Hypertension | 0,30622 | sleep disorders\|hemiplegia\|sleep deprivation\|hearing disorders\|hemianopsia\|body weight\|weight loss\|coma\|psychophysiologic disorders\|hypothermia\|obesity\|urinary bladder, neurogenic\|tremor\|ataxia\|pain\|snoring\|psychomotor agitation\|hypercapnia\|syncope, vasovagal\|seizures\|akathisia, drug-induced\|psychomotor disorders\|syncope\|unconsciousness\|fatigue\|diarrhea\|chorea\|nocturia\|thinness\|amnesia\|flushing\|anorexia\|memory disorders\|headache\|fever\|low back pain\|myoclonus\|confusion\|learning disorders\|vomiting\|dystonia\|aphasia\|vision disorders\|hallucinations\|blindness\|hyperphagia\|dizziness\|nausea\|muscle rigidity\|amnesia, transient global\|paralysis\|color vision defects\|hyperkinesis\|hearing loss\|urinary bladder, overactive\|muscle weakness\|mental retardation\|delirium\|weight gain\|urinary incontinence\|anoxia\|reflex, abnormal\|supranuclear palsy, progressive\|taste disorders | | | |
| Heart Failure | Parkinson Disease | 0,294444 | sleep disorders\|mobility limitation\|sleep deprivation\|edema\|weight loss\|dyspnea\|psychophysiologic disorders\|hypothermia\|obesity\|body weight\|snoring\|hypercapnia\|constipation\|seizures\|vertigo\|syncope\|paresthesia\|muscle hypotonia\|diarrhea\|hyperventilation\|thinness\|muscular atrophy\|myoclonus\|spasm\|memory disorders\|headache\|cheyne-stokes respiration\|fever\|pain\|respiratory sounds\|mental fatigue\|confusion\|asthenia\|vomiting\|hiccup\|aphasia\|coma\|cough\|hypoventilation\|dizziness\|nausea\|anorexia\|dyspepsia\|hemiplegia\|vocal cord paralysis\|nocturia\|muscle weakness\|mental retardation\|fatigue\|weight gain\|anoxia\|reflex, abnormal\|taste disorders | | | |
| Asthma | Stroke | 0,294118 | sleep disorders\|neck pain\|hemiplegia\|hypothermia\|angina pectoris\|edema\|weight loss\|obesity\|proteinuria\|body weight\|pain\|vocal cord paralysis\|hypercapnia\|dyspnea\|seizures\|vertigo\|snoring\|dyslexia\|syncope\|unconsciousness\|paresthesia\|muscle hypotonia\|neuralgia\|muscular atrophy\|myoclonus\|obesity, morbid\|spasm\|blindness, cortical\|memory disorders\|headache\|birth weight\|fever\|apnea\|voice disorders\|mental fatigue\|overweight\|gagging\|paraplegia\|vomiting\|apraxia, ideomotor\|chest pain\|fatigue\|coma\|tremor\|cough\|dizziness\|nausea\|paralysis\|quadriplegia\|hiccup\|respiratory aspiration\|acute coronary syndrome\|hyperkinesis\|respiratory paralysis\|hyperventilation\|muscle weakness\|weight gain\|anoxia\|reflex, abnormal\|taste disorders | | | |
| Alzheimer Disease | Obesity | 0,285714 | sleep disorders\|mobility limitation\|sleep deprivation\|hearing disorders\|diarrhea\|vision disorders\|weight loss\|body weight\|psychophysiologic disorders\|cachexia\|obesity\|urinary bladder, neurogenic\|tremor\|pain\|snoring\|hypercapnia\|hypothermia\|seizures\|perceptual disorders\|psychomotor disorders\|urinary incontinence, urge\|nocturia\|thinness\|muscular atrophy\|anorexia\|memory disorders\|headache\|fever\|low back pain\|learning disorders\|vomiting\|fatigue\|aging, premature\|blindness\|hyperphagia\|dizziness\|nausea\|gait disorders, neurologic\|language development disorders\|hyperkinesis\|urinary bladder, overactive\|muscle weakness\|mental retardation\|delirium\|weight gain\|urinary incontinence\|anoxia\|taste disorders | | | |
| Migraine Disorders | Obesity | 0,285714 | sleep disorders\|sleep deprivation\|hearing disorders\|edema\|hot flashes\|abdominal pain\|pain, postoperative\|weight loss\|psychophysiologic disorders\|hypothermia\|obesity\|body weight\|gastroparesis\|hypercapnia\|constipation\|seizures\|perceptual disorders\|vertigo\|hyperemesis gravidarum\|angina pectoris\|paresthesia\|diarrhea\|blindness\|deafness\|intermittent claudication\|postoperative nausea and vomiting\|anorexia\|back pain\|memory disorders\|headache\|ecchymosis\|pain\|bulimia\|asthenia\|hearing loss, sensorineural\|vomiting\|hyperalgesia\|fatigue\|vision disorders\|tremor\|learning disorders\|dizziness\|nausea\|dyspepsia\|fever\|hyperkinesis\|hyperventilation\|muscle weakness\|mental retardation\|labor pain\|vision, low\|weight gain\|anoxia\|taste disorders | | | |
| Asthma | Dementia | 0,280612 | sleep disorders\|hemiplegia\|sleep deprivation\|angina pectoris\|pain, postoperative\|weight loss\|psychophysiologic disorders\|taste disorders\|body weight\|low back pain\|snoring\|hypercapnia\|hypothermia\|seizures\|vertigo\|dyslexia\|syncope\|unconsciousness\|torticollis\|diarrhea\|thinness\|muscular atrophy\|flushing\|jaundice\|memory disorders\|muscle weakness\|learning disorders\|pain\|myoclonus\|respiratory aspiration\|mental fatigue\|asthenia\|overweight\|paraplegia\|vomiting\|apraxia, ideomotor\|hypergammaglobulinemia\|fatigue\|coma\|tremor\|olfaction disorders\|weight gain\|nausea\|anorexia\|paralysis\|quadriplegia\|fever\|hyperkinesis\|dizziness\|mental retardation\|akathisia, drug-induced\|obesity\|headache\|anoxia\|reflex, abnormal | | | |
| Alzheimer Disease | Myocardial Infarction | 0,267045 | sleep disorders\|hemiplegia\|sleep deprivation\|hemianopsia\|body weight\|weight loss\|coma\|psychophysiologic disorders\|cachexia\|obesity\|tremor\|snoring\|psychomotor agitation\|hypercapnia\|syncope, vasovagal\|seizures\|psychomotor disorders\|syncope\|unconsciousness\|fatigue\|diarrhea\|thinness\|muscular atrophy\|flushing\|memory disorders\|headache\|fever\|pain\|confusion\|dyslexia, acquired\|vomiting\|aphasia\|hallucinations\|blindness\|dizziness\|nausea\|hypothermia\|muscle rigidity\|amnesia, transient global\|paralysis\|hyperkinesis\|urinary bladder, overactive\|muscle weakness\|delirium\|weight gain\|anoxia\|reflex, abnormal | | | |
| Dementia | Heart Failure | 0,259259 | sleep disorders\|mobility limitation\|sleep deprivation\|angina pectoris\|hemianopsia\|deafness\|weight loss\|psychophysiologic disorders\|hypothermia\|taste disorders\|body weight\|snoring\|hypercapnia\|constipation\|seizures\|vertigo\|syncope\|fatigue\|diarrhea\|thinness\|muscular atrophy\|myoclonus\|intermittent claudication\|syncope, vasovagal\|memory disorders\|muscle weakness\|fever\|pain\|tinnitus\|mental fatigue\|confusion\|asthenia\|overweight\|vomiting\|aphasia\|coma\|weight gain\|nausea\|anorexia\|jaundice\|hemiplegia\|nocturia\|dizziness\|mental retardation\|cachexia\|obesity\|headache\|anoxia\|reflex, abnormal | | | |
| Alzheimer Disease | Asthma | 0,254438 | sleep disorders\|hemiplegia\|sleep deprivation\|dizziness\|weight loss\|psychophysiologic disorders\|taste disorders\|body weight\|pain\|snoring\|hypercapnia\|hypothermia\|seizures\|dyslexia\|syncope\|unconsciousness\|diarrhea\|thinness\|muscular atrophy\|flushing\|anorexia\|memory disorders\|headache\|fever\|low back pain\|myoclonus\|learning disorders\|vomiting\|apraxia, ideomotor\|fatigue\|coma\|tremor\|olfaction disorders\|weight gain\|nausea\|paralysis\|hyperkinesis\|muscle weakness\|mental retardation\|akathisia, drug-induced\|obesity\|anoxia\|reflex, abnormal | | | |
| Alzheimer Disease | Heart Failure | 0,24375 | sleep disorders\|mobility limitation\|sleep deprivation\|dizziness\|weight loss\|psychophysiologic disorders\|hypothermia\|taste disorders\|body weight\|snoring\|hypercapnia\|anorexia\|seizures\|syncope\|hemianopsia\|diarrhea\|thinness\|muscular atrophy\|myoclonus\|syncope, vasovagal\|memory disorders\|headache\|fever\|pain\|confusion\|vomiting\|aphasia\|coma\|weight gain\|nausea\|hemiplegia\|fatigue\|nocturia\|muscle weakness\|mental retardation\|cachexia\|obesity\|anoxia\|reflex, abnormal | | | |
| Atherosclerosis | Heart Failure | 0,164948 | birth weight\|albuminuria\|angina pectoris\|abdominal pain\|overweight\|weight loss\|acute coronary syndrome\|angina, unstable\|chest pain\|fatigue\|body weight\|intermittent claudication\|obesity, morbid\|weight gain\|anoxia\|obesity | | | |
| Atherosclerosis | Diabetes Mellitus, Type 2 | 0,149123 | birth weight\|albuminuria\|angina pectoris\|abdominal pain\|body weight\|overweight\|weight loss\|acute coronary syndrome\|angina, unstable\|chest pain\|fatigue\|aging, premature\|intermittent claudication\|obesity, morbid\|weight gain\|anoxia\|obesity | | | |
| Atherosclerosis | Obesity | 0,140351 | birth weight\|albuminuria\|angina pectoris\|abdominal pain\|body weight\|overweight\|weight loss\|acute coronary syndrome\|angina, unstable\|fatigue\|aging, premature\|intermittent claudication\|obesity, morbid\|weight gain\|anoxia\|obesity | | | |
| Atherosclerosis | Myocardial Infarction | 0,132231 | birth weight\|albuminuria\|angina pectoris\|abdominal pain\|overweight\|weight loss\|acute coronary syndrome\|angina, unstable\|chest pain\|fatigue\|body weight\|intermittent claudication\|obesity, morbid\|weight gain\|anoxia\|obesity | | | |
| Asthma | Atherosclerosis | 0,115044 | birth weight\|angina pectoris\|abdominal pain\|overweight\|weight loss\|acute coronary syndrome\|chest pain\|fatigue\|body weight\|obesity, morbid\|weight gain\|anoxia\|obesity | | | |
| Atherosclerosis | Stroke | 0,10559 | birth weight\|albuminuria\|dysarthria\|amaurosis fugax\|overweight\|weight loss\|acute coronary syndrome\|angina, unstable\|chest pain\|fatigue\|angina pectoris\|body weight\|intermittent claudication\|obesity, morbid\|weight gain\|anoxia\|obesity | | | |
| Atherosclerosis | Hypertension | 0,1 | birth weight\|albuminuria\|angina pectoris\|amaurosis fugax\|abdominal pain\|overweight\|weight loss\|acute coronary syndrome\|angina, unstable\|chest pain\|fatigue\|body weight\|intermittent claudication\|obesity, morbid\|weight gain\|anoxia\|obesity | | | |
| Atherosclerosis | Migraine Disorders | 0,085106 | dysarthria\|amaurosis fugax\|abdominal pain\|weight loss\|chest pain\|fatigue\|angina pectoris\|body weight\|intermittent claudication\|weight gain\|anoxia\|obesity | | | |
| Atherosclerosis | Dementia | 0,071429 | dysarthria\|angina pectoris\|body weight\|overweight\|weight loss\|fatigue\|aging, premature\|intermittent claudication\|weight gain\|anoxia\|obesity | | |  |
| Alzheimer Disease | Atherosclerosis | 0,067797 | dysarthria\|aging, premature\|weight loss\|fatigue\|body weight\|weight gain\|anoxia\|obesity |  |  |  |
| Atherosclerosis | Parkinson Disease | 0,045752 | dysarthria\|weight loss\|fatigue\|body weight\|weight gain\|anoxia\|obesity |  |  |  |

**Table S2D.** Scores (Relative Risk) based on the comorbidity of pairs of diseases in the highlighted diseasome cluster.

| Pathophenotype 1 | Pathophenotype 2 | Score |
| --- | --- | --- |
| Atherosclerosis | Hypertension | 12,989699 |
| Alzheimer Disease | Stroke | 8,026102 |
| Hypertension | Stroke | 5,009539 |
| Alzheimer Disease | Parkinson Disease | 4,040591 |
| Migraine Disorders | Stroke | 3,404218 |
| Atherosclerosis | Stroke | 3,228338 |
| Heart Failure | Hypertension | 3,082 |
| Hypertension | Migraine Disorders | 2,847001 |
| Hypertension | Obesity | 2,310182 |
| Parkinson Disease | Stroke | 2,219126 |
| Asthma | Obesity | 2,177355 |
| Atherosclerosis | Heart Failure | 1,763619 |
| Heart Failure | Stroke | 1,619912 |
| Asthma | Heart Failure | 1,60993 |
| Asthma | Migraine Disorders | 1,589369 |
| Asthma | Hypertension | 1,51469 |
| Heart Failure | Obesity | 1,427927 |
| Alzheimer Disease | Atherosclerosis | 1,321639 |
| Migraine Disorders | Obesity | 1,314262 |
| Heart Failure | Parkinson Disease | 1,179529 |
| Atherosclerosis | Obesity | 1,16398 |
| Alzheimer Disease | Heart Failure | 1,121707 |
| Atherosclerosis | Parkinson Disease | 1,114572 |
| Asthma | Atherosclerosis | 1,087526 |
| Alzheimer Disease | Hypertension | 1,058158 |
| Atherosclerosis | Migraine Disorders | 1,02697 |
| Obesity | Stroke | 1,013974 |
| Hypertension | Parkinson Disease | 0,987518 |
| Asthma | Stroke | 0,981042 |
| Migraine Disorders | Parkinson Disease | 0,747548 |
| Asthma | Parkinson Disease | 0,737077 |
| Alzheimer Disease | Migraine Disorders | 0,712727 |
| Alzheimer Disease | Asthma | 0,672374 |
| Heart Failure | Migraine Disorders | 0,652517 |
| Obesity | Parkinson Disease | 0,522947 |
| Alzheimer Disease | Obesity | 0,421937 |

| **Table S3.** Study design. Power analysis. | | | | |  |
| --- | --- | --- | --- | --- | --- |
| tMCAO – BAY58-2776 (30μg/kg) | | | | |  |
|  | *Infarct size (mm^3^)* | | |  | |
|  | *Mean* | *SD* | *N* | *Power for measured difference (%)* | |
| Vehicle | 100,6 | 23,3 | 10 | 84,1 | |
| Treatment | 59,4 | 22,1 | 15 |  |  |
| tMCAO – BAY60-2770 (10μg/kg) | | | | |  |
|  | *Infarct size (mm^3^)* | | |  | |
|  | *Mean* | *SD* | *N* | *Power for measured difference (%)* | |
| *1h post stroke adult animals* | | | | | |
| Vehicle | 112,1 | 28,5 | 19 | 98,5 | |
| Treatment | 61,6 | 23,4 | 18 |  |  |
| *4h post stroke adult animals* | | | | | |
| Vehicle | 128,6 | 20,9 | 10 | 92,6 | |
| Treatment | 67,8 | 23,2 | 5 |  |  |
| *1h post stroke aged animals* | | | | | |
| Vehicle | 60,5 | 39,2 | 8 | 38,2 | |
| Treatment | 20,3 | 16,9 | 8 |  |  |
| tMCAO- All BAY60-2770 treatments 1h post-stroke | | | | |  |
|  | *Infarct size (mm^3^)* | | |  | |
|  | *Mean* | *SD* | *N* | *Power for measured difference (%)* | |
| Vehicle | 96,8 | 39,4 | 27 | 93,8 | |
| Treatment | 48,9 | 28,9 | 26 |  | |
| tMCAO, transient middle cerebral artery occlusion; SD, standard deviation; N, number of animals. We conducted a *post hoc* analysis of power in the different animal groups. For each animal treatment group, a pooled variance of the vehicle and treatment groups was calculated from mean, SD and n-number with n the size of the group and CV the coefficient of variation (SD/Mean) of the group (25). Power was calculated for the measured difference using Russ Lenth’s power software with an alpha of 0.05, the measured effect (%) and the calculated pooled variances | | | | | |

**Table S4.** Degree centrality of diseases across diseasomes.

| Disease | Average degree across all diseasomes | Average degree excluding interactome-based diseasome |
| --- | --- | --- |
| Stroke | 7.25 | 6.56 |
| Alzheimer disease | 5.25 | 4.31 |
| Dementia | 4.75 | 3.43 |
| Atherosclerosis | 4.25 | 3.81 |
| Asthma | 4 | 3.25 |
| Diabetes mellitus, type 2 | 4 | 2.5 |
| Parkinson disease | 4 | 3.25 |
| Heart failure | 3.5 | 3.37 |
| Migraine disorders | 3.25 | 2.31 |
| Myocardial infarction | 3.25 | 2.31 |
| Hypertension | 3 | 3 |
| Obesity | 3 | 2.75 |
